# An epigenome-wide association study of posttraumatic stress disorder in US veterans implicates several new DNA methylation loci

**DOI:** 10.1186/s13148-020-0820-0

**Published:** 2020-03-14

**Authors:** Mark W. Logue, Mark W. Miller, Erika J. Wolf, Bertrand Russ Huber, Filomene G. Morrison, Zhenwei Zhou, Yuanchao Zheng, Alicia K. Smith, Nikolaos P. Daskalakis, Andrew Ratanatharathorn, Monica Uddin, Caroline M. Nievergelt, Allison E. Ashley-Koch, Dewleen G. Baker, Jean C. Beckham, Melanie E. Garrett, Marco P. Boks, Elbert Geuze, Gerald A. Grant, Michael A. Hauser, Ronald C. Kessler, Nathan A. Kimbrel, Adam X. Maihofer, Christine E. Marx, Xue-Jun Qin, Victoria B. Risbrough, Bart P. F. Rutten, Murray B. Stein, Robert J. Ursano, Eric Vermetten, Christiaan H. Vinkers, Erin B. Ware, Annjanette Stone, Steven A. Schichman, Regina E. McGlinchey, William P. Milberg, Jasmeet P. Hayes, Mieke Verfaellie, the Traumatic Stress Brain Study Group, Matthew J. Friedman, Victor E. Alvarez, David Benedek, Christopher Brady, Dianne Cruz, David A. Davis, Ronald S. Duman, Matthew J. Girgenti, Melanie Hardegree, Paul E. Holtzheimer, Terence M. Keane, Neil Kowell, John H. Krystal, Ann McKee, Brian Marx, Deborah Mash, William K. Scott, Thor Stein, Douglas E. Williamson, Keith A. Young

**Affiliations:** 1 grid.410370.1 0000 0004 4657 1992 National Center for PTSD, VA Boston Healthcare System, Boston, MA USA; 2 grid.475010.7 0000 0004 0367 5222 Department of Psychiatry, Boston University School of Medicine, Boston, MA USA; 3 grid.475010.7 0000 0004 0367 5222 ,Biomedical Genetics, Boston University School of Medicine, Boston, MA USA; 4 grid.189504.1 0000 0004 1936 7558 Department of Biostatistics, Boston University School of Public Health, Boston, MA USA; 5 grid.189967.8 0000 0001 0941 6502 Department of Gynecology and Obstetrics, Emory University, Atlanta, GA USA; 6 grid.189967.8 0000 0001 0941 6502 Department of Psychiatry and Behavioral Sciences, Emory University, Atlanta, GA USA; 7 grid.38142.3c 000000041936754X Department of Psychiatry, Harvard Medical School, Boston, MA USA; 8 grid.240206.2 0000 0000 8795 072X McLean Hospital, Belmont, MA USA; 9 Cohen Veterans Bioscience, Cambridge, MA USA; 10 grid.59734.3c 0000 0001 0670 2351 Department of Psychiatry, Icahn School of Medicine at Mount Sinai, New York, NY USA; 11 grid.21729.3f 0000000419368729 Department of Epidemiology, Columbia University, New York, NY USA; 12 grid.170693.a 0000 0001 2353 285X Genomics Program, University of South Florida College of Public Health, Tampa, FL USA; 13 grid.170693.a 0000 0001 2353 285X ,Global Health and Infectious Disease Research Program, University of South Florida College of Public Health, Tampa, FL USA; 14 grid.266100.3 0000 0001 2107 4242 Department of Psychiatry, University of California San Diego, La Jolla, CA USA; 15 grid.410371.0 0000 0004 0419 2708 Center of Excellence for Stress and Mental Health, Veterans Affairs San Diego Healthcare System, San Diego, CA USA; 16 grid.410371.0 0000 0004 0419 2708 Research Service, Veterans Affairs San Diego Healthcare System, San Diego, CA USA; 17 grid.189509.c 0000000100241216 Duke Molecular Physiology Institute, Duke University Medical Center, Durham, NC USA; 18 grid.410371.0 0000 0004 0419 2708 Psychiatry Service, Veterans Affairs San Diego Healthcare System, San Diego, CA USA; 19 grid.26009.3d 0000 0004 1936 7961 Department of Psychiatry and Behavioral Sciences, Duke University, Durham, NC USA; 20 grid.410332.7 0000 0004 0419 9846 Research, Durham VA Medical Center, Durham, NC USA; 21 grid.281208.1 0000 0004 0419 3073 Genetics Research Laboratory, VA Mid-Atlantic Mental Illness Research, Education, and Clinical Center (MIRECC), Durham, NC USA; 22 grid.7692.a 0000000090126352 Department of Psychiatry, UMC Utrecht Brain Center, Utrecht, Utrecht Netherlands; 23 Brain Research and Innovation Centre, Netherlands Ministry of Defence, Utrecht, Utrecht Netherlands; 24 grid.240952.8 0000000087342732 Department of Neurosurgery, Stanford University Medical Center, Stanford, CA USA; 25 grid.38142.3c 000000041936754X Department of Health Care Policy, Harvard Medical School, Boston, MA USA; 26 grid.26009.3d 0000 0004 1936 7961 Duke Molecular Physiology Institute, Duke University, Durham, NC USA; 27 grid.21925.3d 0000 0004 1936 9000 Department of Critical Care Medicine, Neurology, and Neurosurgery, University of Pittsburgh, Pittsburgh, PA USA; 28 grid.189509.c 0000000100241216 Department of Psychiatry & Behavioral Sciences, Duke University Medical Center, Durham, NC USA; 29 grid.412966.e 0000 0004 0480 1382 School for Mental Health and Neuroscience, Department of Psychiatry and Neuropsychology, Maastricht Universitair Medisch Centrum, Maastricht, Limburg Netherlands; 30 grid.410371.0 0000 0004 0419 2708 Million Veteran Program, Veterans Affairs San Diego Healthcare System, San Diego, CA USA; 31 grid.265436.0 0000 0001 0421 5525 Department of Psychiatry, Uniformed Services University, Bethesda, MD USA; 32 Arq, Psychotrauma Reseach Expert Group, Diemen, NH Netherlands; 33 grid.10419.3d 0000000089452978 Department of Psychiatry, Leiden University Medical Center, Leiden, ZH Netherlands; 34 Netherlands Defense Department, Research Center, Utrecht, UT Netherlands; 35 grid.137628.9 0000 0004 1936 8753 Department of Psychiatry, New York University School of Medicine, New York, NY USA; 36 Department of Anatomy and Neurosciences, Amsterdam UMC (location VUmc), Amsterdam, Holland Netherlands; 37 Department of Psychiatry, Amsterdam UMC (location VUmc), Amsterdam, Holland Netherlands; 38 grid.214458.e 0000000086837370 Institute for Social Research, Survey Research Center, University of Michigan, Michigan, MI USA; 39 grid.413916.8 0000 0004 0419 1545 Pharmacogenomics Analysis Laboratory, Research Service, Central Arkansas Veterans Healthcare System, Little Rock, AR USA; 40 grid.410370.1 0000 0004 4657 1992 Geriatric Research Educational and Clinical Center and Translational Research Center for TBI and Stress Disorders, VA Boston Health Care System, Boston, MA USA; 41 grid.261331.4 0000 0001 2285 7943 Department of Psychology and Chronic Brain Injury Program, The Ohio State University, Columbus, OH USA; 42 grid.475010.7 0000 0004 0367 5222 Memory Disorders Research Center, VA Boston Healthcare System and Boston University School of Medicine, Boston, MA USA

## Abstract

**Background:**

Previous studies using candidate gene and genome-wide approaches have identified epigenetic changes in DNA methylation (DNAm) associated with posttraumatic stress disorder (PTSD).

**Methods:**

In this study, we performed an EWAS of PTSD in a cohort of Veterans (*n* = 378 lifetime PTSD cases and 135 controls) from the Translational Research Center for TBI and Stress Disorders (TRACTS) cohort assessed using the Illumina EPIC Methylation BeadChip which assesses DNAm at more than 850,000 sites throughout the genome. Our model included covariates for ancestry, cell heterogeneity, sex, age, and a smoking score based on DNAm at 39 smoking-associated CpGs. We also examined in EPIC-based DNAm data generated from pre-frontal cortex (PFC) tissue from the National PTSD Brain Bank (*n* = 72).

**Results:**

The analysis of blood samples yielded one genome-wide significant association with PTSD at cg19534438 in the gene *G0S2* (*p* = 1.19 × 10^-7^, *p*_adj_ = 0.048). This association was replicated in an independent PGC-PTSD-EWAS consortium meta-analysis of military cohorts (*p* = 0.0024). We also observed association with the smoking-related locus cg05575921 in *AHRR* despite inclusion of a methylation-based smoking score covariate (*p* = 9.16 × 10^-6^), which replicates a previously observed PGC-PTSD-EWAS association (Smith et al. 2019), and yields evidence consistent with a smoking-independent effect. The top 100 EWAS loci were then examined in the PFC data. One of the blood-based PTSD loci, cg04130728 in *CHST11*, which was in the top 10 loci in blood, but which was not genome-wide significant, was significantly associated with PTSD in brain tissue (in blood *p* = 1.19 × 10^-5^, *p*_adj_ = 0.60, in brain, *p* = 0.00032 with the same direction of effect). Gene set enrichment analysis of the top 500 EWAS loci yielded several significant overlapping GO terms involved in pathogen response, including “Response to lipopolysaccharide” (*p* = 6.97 × 10^-6^, *p*_adj_ = 0.042).

**Conclusions:**

The cross replication observed in independent cohorts is evidence that DNA methylation in peripheral tissue can yield consistent and replicable PTSD associations, and our results also suggest that that some PTSD associations observed in peripheral tissue may mirror associations in the brain.

## Introduction

Genetic studies of posttraumatic stress disorder (PTSD) diatheses conducted to date have focused primarily on identifying DNA variants (e.g., single-nucleotide polymorphisms, SNPs) that confer risk for the development of the disorder through candidate gene or genome-wide association studies (GWASs; see, e.g., [1, 2]). More recently, studies have also examined differences between PTSD cases and controls in patterns of gene expression [3] and/or DNA methylation (DNAm [4–6];). DNAm studies involve measurement of a methyl group on the DNA strand at a cytosine-phosphate-guanine (CpG) site, and when this is present in the promoter region of a gene, DNAm tends to be negatively correlated with the expression of the gene. DNAm across the genome can be influenced by a host of genetic and developmental mechanisms, health conditions, and environmental factors ranging from toxin exposure to stress, and it is widely hypothesized to be a mechanism that mediates the effects of trauma exposure on gene expression [7, 8].

Hypothesis and mechanism-focused candidate gene studies have identified PTSD-related differences in DNAm levels in genes associated with the hypothalamic-pituitary-adrenal (HPA) axis (e.g., *ADCYAP1* [9]*, FKBP5* [10], and *NR3C1* [11]), inflammation (e.g., *BDNF* [4], *HTR2A* [12] and *IL-18* [13]), and neurotransmission (e.g., *BDNF* [4], *HTR2A* [14], and *HTR3A* [15]). Epigenome-wide association studies (EWASs), on the other hand, take a hypothesis-free approach to identifying DNAm loci from across the genome that are statistically associated with the phenotype of interest. To date, only five published PTSD EWASs have reported single-site associations that survived epigenome-wide multiple-testing correction. First, using a DNAm bead chip that interrogated ~ 27K loci in a sample of 100 subjects from an urban community cohort, Smith et al. (2011) reported false discovery rate (FDR)-corrected differences between PTSD cases and controls at loci in 5 genes (*ACP5*, *ANXA2*, *CLEC9A*, *TLR8*, and *TPR*) [4]. Second, in a study that used a more comprehensive platform measuring methylation at ~ 850K loci in samples from 96 Australian Vietnam veterans, Mehta et al. (2017) found genome-wide significant associations between DNAm and PTSD in four genes (*BRSK1*, *DOCK2*, *LCN8*, and *NGF*) and in one intergenic locus [5]. Rutten et al. (2018) examined pre- to post-deployment changes in DNAm in a cohort of 93 soldiers using a ~ 450K platform and found 17 loci in or near 8 genes that were associated with increasing PTSD symptoms over time [6]. In that study, replication analyses in a similar pre- and post-deployment cohort of 98 soldiers also showed nominal support for findings in 3 genes (*HIST1H2APS2*, *RNF39*, and *ZFP57*). In an EWAS of methylation in sperm cells from a cohort of Veterans (16 with PTSD and 22 controls), Mehta et al. identified three loci reaching genome-wide significance: two intergenic loci and a CpG in *CCDC88C* [16]. This could point to a possible role of these loci in the inter-generational transmission of the effects of trauma. Finally, Smith et al. [17] recently reported results of the largest PTSD EWAS conducted to date based on a meta-analysis of *n* = 1896 participants from 10 cohorts with methylation assessed at ~ 450K loci. Ten loci achieved genome-wide significance, the most significant of which, cg05575921 (*p* = 4.27 × 10^-11^, FDR = 2.15 × 10^-5^), was located in the smoking-associated gene *AHRR*. Many of the EWASs of PTSD have also used Gene Set Enrichment Analysis or Functional Network Analysis of genome-wide DNAm as a follow up to their genome-wide association analyses. Significant enrichment has been observed for a number of pathways/biological processes with plausible relevance to PTSD including, most notably, inflammation and immune function, HPA axis and glucocorticoid signaling, neurogenesis and neurotransmission, circadian rhythms, and cell adhesion [5, 6, 18–20].

To summarize, prior studies of DNAm associations with PTSD have yielded potentially important insights into the epigenetics of PTSD; however, with several noteworthy limitations. All of them were based on DNAm from peripheral samples (usually blood). Though potentially useful for the development of diagnostic biomarkers, blood samples provide only indirect evidence of epigenetic processes in the brain. In addition, most of the sample sizes studied to date have been modest, and the few EWAS-significant associations that have been reported have been accompanied by limited evidence of replication.

Another major consideration involves the influence of potential confounding variables such as cigarette smoking in PTSD studies. Numerous studies have shown the methylome to be exquisitely sensitive to the effects of smoking. The largest EWAS of cigarette smoking conducted to date by Joehanes et al. (*N* ~ 16K), identified 18760 CpGs on 7201 genes that were differentially methylated in current versus never smokers [21]. Results of that study confirmed numerous prior reports of associations between smoking and DNAm in several genes including, most notably, *AHRR*, *F2RL3*, and *RARA*. Because cross-sectional epidemiological studies do not permit inferences about whether DNAm associations are causes, consequences, or effects of third variables, Li et al. (2018) used a genetically informative twin cohort to examine the heritability of the smoking-associated DNAm loci and found strong evidence that most of the observed epigenetic associations were attributable to the effects of cigarette use [22]. These findings are highly relevant to the epigenetics of PTSD because PTSD samples, especially from veteran cohorts, tend to have an elevated prevalence of cigarette smoking relative to the general population [23]. The *AHRR* locus cg05575921 that was associated with PTSD in the Smith et al. EWAS [17] was also highly significant in both the Joehanes et al. EWAS of smoking [21] and the Li et al EWAS of smoking [22], and in the latter, was the most significant locus. Based on this, Smith et al. also performed analyses stratified by smoking status and found that the association between PTSD and cg05575921 was strongest among non-smokers suggesting an association between PTSD and *AHRR* independent of smoking.

The aim of this study was to identify individual CpG sites and/or sets of genes associated with PTSD using DNAm data from a veteran cohort and a newly-established United States (US) Department of Veterans Affairs (VA) National PTSD Brain Bank [24]. Given the robust associations between smoking and DNAm observed in prior studies, we addressed this important confound by computing a DNAm smoking score based on the top loci from the Li et al. (2018) study and including it as a covariate in our analyses. The primary analysis was a “Discovery” EWAS of lifetime PTSD diagnosis in DNA from whole blood drawn from a cohort of veterans of the post-9/11 conflicts in Iraq and Afghanistan. We then evaluated evidence for replication of the top EWAS results in (a) an analysis of veterans performed as part of the Smith et al. EWAS, and (b) postmortem pre-frontal cortical tissue from the National PTSD Brain Bank. Finally, we examined candidate gene regions and candidate CpGs implicated in previous PTSD epigenetic studies.

## Materials and methods

### Discovery Cohort

The Discovery Cohort was comprised of veterans recruited by the Translational Research Center for TBI and Stress Disorders (TRACTS), a Department of Veterans Affairs Rehabilitation Research and Development (RR&D) Traumatic Brain Injury Center of Excellence at VA Boston Healthcare System. After applying a quality control pipeline and excluding participants with missing data (see Additional file 1), 541 veterans (*n* = 378 cases and 135 controls) with DNAm data were available for analyses. Demographic characteristics for all three cohorts are listed in Additional file 1: Table S1. The collection of Discovery Cohort data was performed with the approval of a Department of Veterans Affairs human subjects review board and all subjects provided written informed consent.

### Consortium Replication Cohort

We examined results from the recent Smith et al. Consortium EWAS of current PTSD. As it was the closest match to our Discovery Cohort, we requested results from a military cohort meta-analysis. It included 1351 subjects from 7 cohorts: Army Study to Assess Risk and Resilience in Servicemembers (Army STARRS) [25], Marine Resiliency Study (MRS) [26, 27], Prospective Research in Stress-related Military Operations (PRISMO) [28, 29], The African American and the European American cohorts from the Mid-Atlantic Mental Illness Research Education and Clinical Center PTSD Study (VA-M-AA and VA-M-EA) [30], the Injury and Traumatic Stress study (INTRuST; see e.g., [31–33]), and the VA Boston Healthcare System National Center for PTSD (VA-NCPTSD) cohort [1]. In the replication cohort analysis, 42% were current PTSD cases, all of which were assessed with Illumina Infinium HumanMethylation450 BeadChip (450K BeadChips; see Smith et al 2019 for details). As it was not available for many participating cohorts, lifetime PTSD was not analyzed.

### PTSD Brain Bank

DNA was extracted from the ventromedial prefrontal cortex (vmPFC; Brodmann area 12/32) and dorsolateral prefrontal cortex (dlPFC, Brodmann area 9/46) from 42 PTSD cases and 30 controls. These regions were selected based on findings from functional and structural imaging studies of PTSD suggesting their involvement in the neurobiology of the disorder (see e.g., [34]). A detailed description of the donor identification, post-mortem diagnostic assessment procedures, and tissue extraction and processing is presented elsewhere [24, 35] and in Additional file 1.

### Generation of genotype and DNAm data

Genome-wide genotype data were used to generate principal components (PCs) to control for ancestral heterogeneity in each analysis. Genotypes for the Discovery and Brain Bank Cohorts were based on data from Illumina (San Diego CA) HumanOmni2.5-8 BeadChips as described previously [1, 36] and in the Supplementary Methods (Additional file 1). DNAm was assessed in the Discovery and the Brain Bank Cohorts using the Illumina EPIC 850K BeadChips. An EWAS consortium-derived pipeline was used to clean the DNAm data from both cohorts [37]. Additional information about the quality control (QC) pipeline is available in Additional file 1.

### Data analyses

Analyses examining associations between DNAm and lifetime PTSD case/control status in the Discovery Cohort were performed with linear models as automated in the Bioconductor limma (Linear Models for Microarray Data) package [38] with the base 2 logit-transformed methylated proportion as the response and PTSD diagnosis as a predictor. Note that we will avoid the use of the term “beta” to refer to coefficient estimates from linear models throughout to avoid confusion with the term beta as it applies to DNAm studies, where it is used to refer to the proportion of DNAm at a given locus. Each limma model included principal components for ancestry, age, sex, estimates of white blood cell proportions, and a DNAm-based “smoking score” as covariates. The latter was based on effect-size estimates for the top-39 probes from a recent smoking EWAS [22]. This score showed highly significant association with self-reported smoking in both the Discovery Cohort and in the VA-NCPTSD cohort (*p* < 2.2 × 10^-16^ in both cohorts), and was significantly correlated with the number of cigarettes per day in the Discovery Cohort (*r* = 0.52, *p* < 2.2 × 10^-16^). See Supplementary Methods (Additional file 1) for details about computation of the smoking score.

For the EWAS in the Discovery Cohort, we computed false discovery rate [39] corrected *p*-values, also known as Q-values, to control for multiple testing (denoted “*p*_adj_”). We then examined the top 100 associated sites from the EWAS in the 450K consortium results and also in the Brain Bank Cohort. In cases in which the observed top associated EPIC locus was not assayed by the 450K BeadChip, when available, we examined correlated “proxy sites” for evidence of replication. A proxy site was defined as a CpG assayed by both EPIC and 450K BeadChips within 5000 bp of a peak EPIC locus that was significantly correlated (*p* < 0.05) to the peak EPIC site. When we observed multiple correlated sites to a peak EPIC locus, we took the one with the highest correlation as the proxy. The genes corresponding to the top 500 sites from the Discovery EWAS were examined for enrichment of specific gene ontology (GO) term categories using the gometh function from the R missMethyl package [40]. This function is an extension of the GOseq method [41] which explicitly models the relationship between the number of CpG sites measured within a gene and the probability of that gene appearing within the target list, hence avoiding one of the sources of bias which can influence gene set enrichment analyses. Next, we examined candidate genes and CpG sites previously implicated in studies of DNAm and PTSD. We began by evaluating the significance of the 41 previously implicated CpG sites in blood-based studies of PTSD [4–6, 9, 12, 19, 36, 42, 43] (listed in Additional file 1: Table S2) in the limma EWAS results. Then, we performed a candidate gene examination of 36 previously implicated genes, by examining all sites within a gene from the limma output for association with PTSD using a gene-wide FDR correction based on the number of probes in each gene.

Post-hoc analyses, including evaluation of the role of smoking in the top EWAS associations and also the effects of other potential confounders (depression, depression severity, selective serotonin reuptake inhibitor use, and alcohol use) in the Discovery Cohort, were performed in R using the standard package for linear modeling (lm). As DNAm values can be influenced by nearby SNPs, we also examined the possibility of SNP effects for our top-associated loci. We ran a model similar to what was performed in limma above, but which included as a covariate imputed SNP dosage of any nearby genomic variants noted in the Illumina Annotation with minor allele frequency > 5%. See the Supplementary Materials (Additional file 1) for a more complete description of these analyses.

When analyzing the data from the PTSD brain bank, both regions (vmPFC and dlPFC) were analyzed jointly to increase power, reduce multiple testing, and to focus our attention on methylation differences that are consistent across the PFC. A linear mixed model was used which included a random effect to adjust for the correlations of DNAm between the two brain regions in the same subject. The model for the PFC included covariates for age, sex, ancestry PCs, and proportion of neurons as estimated from the methylation data [44]. The smoking score when applied to DNAm data from the PFC was found not to correlate with smoking, and indeed, an investigation of smoking in the PFC did not yield any EWAS-significant smoking loci (see Additional file 1 for details). This is consistent with a smaller effect of smoking on methylation in the PFC compared to blood. However, given the modest sample size, we could not exclude such an effect, and smoking as determined by family report and medical history was included as a covariate in the PFC analyses. Although the Brain Bank Cohort was used here primarily to replicate the results from the EWAS performed in blood, we computed the results for the entire genome, so that we could examine the genome-wide distribution of *p*-values for inflated significance. Follow-up analyses in limma examined the dlPFC and vmPFC regions separately to explore whether significant associations were jointly observed across regions.

As we have elsewhere shown that a large proportion of low variation probes have poor correlation across chips due to a low signal-to-noise ratio [45], we excluded probes from the EWAS and candidate gene analyses when the range of the proportion of DNAm was < 0.10; these were primarily sites where the DNAm proportion was < 0.10 for all subjects or the DNAm proportion was > 0.90 for all subjects. This criterion excluded 417,270 of the 819,877 probes passing quality filters (see Additional file 1 for details), leaving 402,607 sites in the blood-based EWAS. However, these low variation probes were analyzed in limma and retained for the purposes of replication of previously reported loci.

## Results

### Discovery Cohort EWAS

**Fig. 1:**
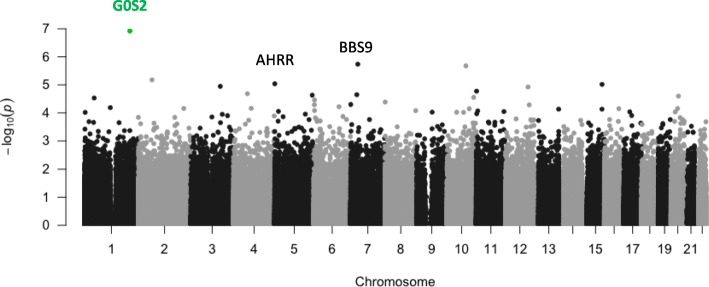
Manhattan plot of an epigenome-wide association study of PTSD in US Veterans, the one EWAS significant locus at *G0S2* is highlighted in green

**Fig. 2:**
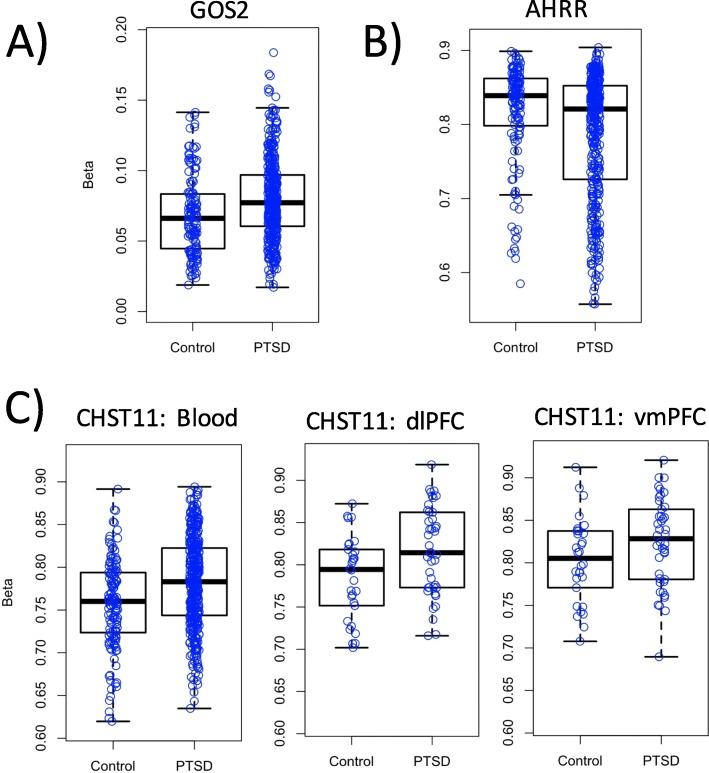
Box/Scatter plot of the proportion of DNAm (Beta) for (**a**) cg19534438, the genome-wide significant locus in *G0S2*, (**b**) cg05575921, the smoking- and PTSD-associated locus in *AHRR*, and (**c**) the *CHST11* peak locus from the EWAS of whole-blood samples that was corrected significant in the analysis of tissue from the PFC, with blood, dlPFC, and vmPFC methylation plotted separately.

There was no evidence of inflation for Discovery EWAS analysis of 402,607 probes (lambda = 1.066; Additional file 1: Fig. S1). Manhattan plots (− log10 *p*-values for each locus plotted relative to their genomic position) are presented in Fig. 1, and the top 10 most strongly associated loci are listed in Table 1. One epigenome-wide significant association was found involving probe cg19534438 in the G0/G1 Switch 2 gene (*G0S2*) on chromosome 1 (coefficient = 0.34, *p* = 1.19 × 10^-7^, *p*_adj_ = 0.048; see Fig. 2a). The other top 10 results included probes in *BBS9, RCCD1*, *NCK1*, *CHST11*, *TMLHE*, 3 intergenic loci, and *AHRR* (see Additional file 1: Fig. S2). The *AHRR* result, involving cg05575921 (Fig. 2b), was particularly noteworthy because this probe was one of the 39 used to compute the DNAm smoking score that was included as a covariate in the EWAS. To clarify the nature of this association and determine if it was an artifact of comorbid cigarette use in the PTSD cases, we performed post-hoc analyses examining the association between this *AHRR* probe, self-reported smoking, the smoking score, and PTSD under various conditions including (a) a model of PTSD with the smoking score excluded, and (b) a model of PTSD with the smoking score included but with cg05575921 excluded from score calculation. In both cases, the association between cg05575921 and PTSD remained robust (*p* < 10^-4^; see Supplementary Results and Additional file 1:Tables S3 and S4 for details). As Smith et al. reported that the association between cg05575921 and PTSD was more significant in non-smokers than in smokers, we performed a similar follow-up analysis of subjects segregated by smoking status. Although cg05575921 was not significant in either smokers or non-smokers, perhaps due to the reduction of sample size, there is a more substantial (negative) effect size estimate for cg05575921 in the non-smokers than the smokers (in non-smokers, coefficient = − 0.13, *p* = 0.059; in smokers, coefficient =− 0.048, *p* = 0.79). Post-hoc analyses also showed that the EWAS-significant association involving cg19534438 in *G0S2*, was not affected by the inclusion or exclusion of self-reported smoking or the DNAm-based smoking score in the model (Additional file 1: Table S4).

**Table 1:** Top 10 strongest associations from the Discovery Cohort EWAS (*n* = 378 cases and 135 controls)

Gene	ID	Coefficient	*p*-value	*p*_adj_
*G0S2*	cg19534438	0.34	**1.19E** − **7**	**0.048**
*BBS9*	cg20152234	0.18	1.83E − 6	0.28
Intergenic	cg11504264	0.24	2.09E − 6	0.28
Intergenic	cg08000207	− 0.35	6.64E − 6	0.60
*AHRR*	cg05575921	− 0.13	9.16E − 6	0.60
*RCCD1*	cg25526519	− 0.16	9.66E − 6	0.60
*NCK1*	cg09423651	− 0.53	1.13E − 5	0.60
*CHST11*	cg04130728	0.15	1.19E − 5	0.60
*TMLHE*	cg12115116	0.25	1.53E − 5	0.68
Intergenic	cg20974659	0.11	1.68E − 5	0.68

### Consortium Military Replication Cohort

**Fig. 3:**
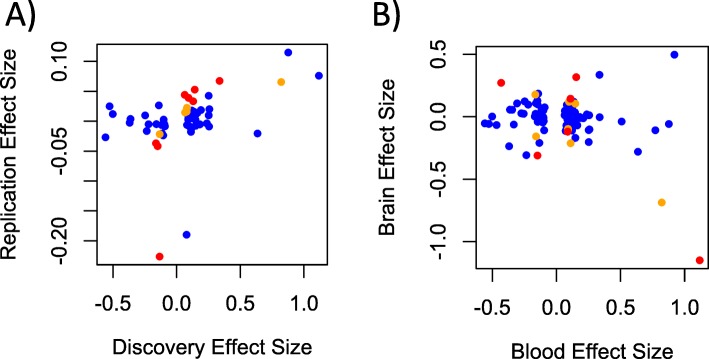
Effect size estimates compared between (**a**) the Discovery and the Consortium Military Replication Cohorts, and (**b**) the Discovery (blood) and the analysis of methylation in the PFC (brain). Loci with 0.05 < *p* < 0.10 are plotted in orange, and loci with *p* < 0.05 are plotted in red

Of the top 100 loci from our EWAS, consortium 450K meta-analysis results were available for 54, and proxies were available for an additional 6. We observed 8 nominally significant associations in the replication cohort, more than would be expected under the null (*p* = 0.0098 based on a binomial distribution with 5% chance of success). Across the 60 loci, the effect size estimates were significantly correlated (*r* = 0.39, *p* = 0.0022, see Fig. 3a). The correlation estimate was higher for the *n* = 14 loci with *p* < 0.10 in the Replication Cohort (orange and red points in Fig. 3a; *r* = 0.58, *p* = 0.030) and in the 8 loci with *p* < 0.05 in the Replication Cohort (red points in Fig. 3a; *r* = 0.72, *p* = 0.043). All loci with *p* < 0.10 in the Replication Cohort had the same direction of effect as the Discovery Cohort, which is highly unlikely under the null hypothesis of a 50% chance of agreement (*p* = 0.00012). Five of the loci assessed in the Replication Cohort remained significant after correcting for the 60 loci examined (Table 2). Unsurprisingly, the *AHRR* locus noted in our analysis was significant in the Consortium Military analysis. Two related genes, *APBA1* and *APBA2* also replicated. The *APBA1* association was based on cg13580827, a proxy for the EPIC-only probe cg06826552, while the *APBA2* locus was present on both chips. The *G0S2* locus, which was genome-wide significant in our EWAS, was likewise associated with PTSD in the Consortium Military Replication Cohort (*p* = 9.18 × 10^-4^, *p*_adj_ = 0.014). Finally, cg25526519 in the LOC285696 gene also replicated (*p* = 0.0033, *p*_adj_ = 0.040).

**Table 2:** Probes from the top 100 results in the Discovery EWAS that were significantly associated with PTSD in the Consortium Military Meta-Analysis Replication Cohort

Cohort		Discovery *n* = 513		Replication *n* = 1351		
Gene	ID	Coef.	*p*	Coef.	*p*	*p*_adj_
*AHRR*	cg05575921	− 0.13	9.16E − 6	− 0.23	1.15E − 10	6.90E − 9
*APBA2*	cg27184903	0.063	0.00023	0.044	4.51E - 5	0.0013
*APBA1*	cg06826552/ proxy cg13580827	− 0.15/ − 0.060	9.43E − 5/ 0.0070	NA/ − 0.042	NA/ 6.37E − 5	NA/ 0.0013
*G0S2*	cg19534438	0.34	1.19E − 7	0.067	0.00092	0.014
*LOC285696*	cg23987134	0.093	8.82E − 5	0.039	0.0033	0.040

### Brain Bank Cohort results

When we compared the effect size estimates across the Discovery Cohort top 100 EWAS loci in the Brain Bank PFC samples, we did not observe the same correspondence observed between the two blood-based cohorts. In fact, we observed a significant negative correlation (*r* = − 0.30, *p* = 0.0022, see Fig. 3b). This was driven primarily by cg12186981 in *OR2AG1*, which, of the top 100 blood results we attempted to replicate in brain, had the largest effect size estimated in both blood and brain, but with an opposite direction (in blood: effect size = 1.12, *p* = 8.49 × 10^-5^, in brain effect size = − 1.15, *p* = 0.033). When this point was excluded, the correlation in effect size estimates across blood and brain was still negative, but not significant (*r* = − 0.096, *p* = 0.334). Of the 100 loci examined in the PFC analysis, one, cg04130728 in the *CHST11* gene, survived multiple-testing correction (Table 3). This probe was among the top 10 results in the Discovery Cohort, and the direction of effect was the same across the two cohorts. Analysis of the dlPFC and vmPFC separately in limma indicated that the associations observed with *CHST11* were consistent across both brain regions (in dlPFC, coefficient = 0.32, *p* = 0.0034; in vmPFC, coefficient = 0.30, *p* = 0.011, see Fig. 2c). Information on this locus in the Replication Cohort was not available, as it was not assessed on the 450K chip, nor were there any correlated proxy sites. This analysis also revealed two nominally significant associations with the same direction of effect across the blood and brain samples, namely, cg11339964 in *FBXL7*, and cg12186981 in *PHACTR* (Additional file 1: Fig. S3). Three other nominally significant associations had opposite directions of effect across the discovery and brain bank samples. Genome-wide analysis of the PFC samples did not yield any evidence of inflated significance (Additional file 1: Fig. S4).

**Table 3:** Probes from the top 100 results in the Discovery EWAS that were nominally associated with PTSD in a combined PFC analysis of dlPFC and vmPFC samples from the Brain Bank Cohort

		Discovery *n* = 513		Brain bank *n* = 72		
Gene	ID	Coefficient	*p*	Coefficient	*p*	*p*_adj_
*CHST11*	cg04130728	**0.15**	1.19E − 05	**0.32**	**0.00032**	**0.032**
*FBXL7*	cg11339964	**0.11**	1.97E − 04	**0.14**	0.020	0.66
*PHACTR*	cg19686983	− **0.15**	0.00017	− **0.31**	0.030	0.66
*OR2AG1*	cg12186981	1.12	8.49E − 05	− 1.15	0.033	0.66
*C12orf34*	cg02742775	0.087	5.18E − 05	− 0.12	0.042	0.66
*F9*	cg03155646	− 0.43	4.32E − 05	0.27	0.045	0.66

### Gene set enrichment analysis

The gene set enrichment analyses of the top 500 loci from the EWAS of the Discovery Cohort revealed five FDR-corrected significant GO terms (see Table 4). Three of the five (GO:0032496: response to lipopolysaccharide, GO:0002237: response to molecule of bacterial origin, and GO:0071216: cellular response to biotic stimulus) involve pathogen response. The genes in these three categories overlap substantially, with each containing *PPARGC1A*, *TICAM1*, *TICAM2*, *IL1B*, *MEF2C*, *ABCB4*, *PRKCE*, *HAMP*, *RARA*, *TFPI*, *VDR*, *CDC73*, and *TNIP3*. The other two significant GO terms were GO:0030374: ligand-dependent nuclear receptor transcription coactivator activity and GO:0003416: endochondral bone growth.

**Table 4:** Significant enrichment of GO terms in the top 500 sites from the Discovery Cohort EWAS of PTSD

Term	Ont	*N*	DE	*p*	*p*_adj_	Genes
GO:0032496: response to lipopolysaccharide	BP	322	19	6.97E − 06	0.042	*PPARGC1A, CNR2, CPS1, TICAM1, ELANE, FGFR2, FOXP1, TICAM2, IL1B, MEF2C, ABCB4, PRKCE, ERBIN, HAMP, RARA, TFPI, VDR, CDC73, TNIP3*
GO:0030374: ligand-dependent nuclear receptor transcription coactivator activity	MF	69	9	1.12E − 05	0.042	*PPARGC1A, NCOA7, PPARG, PRKCB, RARA, RARB, RORA, VDR, MED12*
GO:0002237:response to molecule of bacterial origin	BP	339	19	1.42E − 05	0.042	*PPARGC1A, CNR2, CPS1, TICAM1, ELANE, FGFR2, FOXP1, TICAM2, IL1B, MEF2C, ABCB4, PRKCE, ERBIN, HAMP, RARA, TFPI, VDR, CDC73, TNIP3*
GO:0071216: cellular response to biotic stimulus	BP	202	14	1.71E − 05	0.042	*PPARGC1A, TICAM1, TICAM2, IL1B, MEF2C, ABCB4, PRKCE, HAMP, RARA, TFPI, VDR, WFS1, CDC73, TNIP3*
GO:0003416: endochondral bone growth	BP	22	6	1.75E − 05	0.042	*FGFR2, POC1A, MSX2, BNC2, RARA, RARB*

### Candidate probes and candidate gene regions

Of the 51 CpG sites with prior reports of association with PTSD (Additional file 1: Table S5), only the *AHRR* locus reported by Smith et al. survived correction for multiple testing (*p*_adj_ = 0.00047), which was not surprising given the fact that cg05575921 was near genome-wide significant in our EWAS. Two additional loci showed nominally significant associations in the Discovery Cohort: cg20098659 in *CLEC9A* [4] (coefficient = 0.087, *p* = 0.0098, *p*_adj_ = 0.25) and cg02357741 in *BRSK1* [5] (coefficient = 0.054, *p* = 0.045, *p*_adj_ = 0.63). When we examined all of the probes in each of the previously implicated genes (Additional file 1: Table S6), 3 genes showed associations with PTSD that withstood corrections for the number of probes in the respective gene: (a) *AHRR* (coefficient = − 0.13, *p* = 9.16 × 10^-6^, *p*_adj_ = 0.00082); (b) *CLEC9A*, where the most significant association was with cg02930518 (coefficient = 0.089, *p* = 0.0067, *p*_adj_ = 0.040); and (c) *COL9A3*, where the most significant locus was cg08021508 (coefficient = − 0.16, *p* = 0.00066, *p*_adj_ = 0.025). However, only the *AHRR* association would survive a further adjustment for the 41 previously implicated candidate genes examined.

### Potential confounders

See the Supplementary Results and Additional file 1: Table S7 for an examination of potential confouders including depression, anti-depressant use, alcohol-use disorders, and SNP effects. All of the CpGs in Table 1 remain significant after inclusion of each of these covariates. Similarly, the three loci in Tables 1, 2, 3 with nearby > 5% MAF SNPs remained significant after adjustment for SNP effects. We noted that rs3817870 was a methylation quantitative trait locus (meQTL) for cg19534438 in *G0S2* (*p* = 0.023) and rs1550637 was a meQTL for cg25526519 in *RCCD1*, but that these SNPs were not confounded with the PTSD associations observed at these two loci.

## Discussion

We performed an EWAS of lifetime PTSD diagnosis using whole blood samples from a cohort of trauma-exposed Veterans of the post-9/11 conflicts. We then evaluated evidence for replication using (a) results from a consortium-based meta-analysis of PTSD using military cohorts and (b) DNAm from PFC from a newly established PTSD brain bank. We observed one epigenome-wide significant association involving cg19534438 in the *G0/G1 Switch 2* (*G0S2*) gene. Methylation at this locus showed a positive association with PTSD diagnosis. This was replicated in the consortium meta-analysis with the same direction of effect (*p* = 0.0024, *p*_adj_ = 0.037). There is some prior evidence for a G0S2-PTSD link. *In vivo* and *in vitro* studies have shown cortisol to suppress G0S2 [46]. Two prior studies have linked *G0S2* expression to PTSD. Specifically, using genome-wide expression profiling in a predator-scent stress animal model of PTSD, Daskalakis et al. (2014) found that *G0S2* expression was downregulated in both the amygdala and hippocampus of female rats [47]. Similarly, Bam et al. (2016) identified *G0S2* as the most downregulated gene in blood from a genome-wide analysis of RNAseq data from a small cohort of PTSD patients (*n* = 5) and controls (*n* = 5) [48]. The G0S2 protein is well-known for its role in regulating lipid metabolism where it serves as a negative regulator of lipolysis [49, 50]. It has been implicated in mechanisms of obesity, diabetes, aging, and cancer, and linked to gene networks involved in apoptosis, cell communication, and cell death [51]. Because of this, future investigations could examine a potential role of *G0S2* in the well-established link between PTSD and metabolic disorders [52, 53].

Although we did not see significant evidence of association between cg19534438 and PTSD in the brain bank data, we note that there is evidence in two online databases that DNAm levels at this locus are correlated between blood and brain. The first database is from the University of Essex (http://epigenetics.essex.ac.uk/bloodbrain/) [54] and includes dual assessment of blood and tissue from four different brain regions: PFC, entorhinal cortex, superior temporal gyrus, and cerebellum with methylation assessed using the 450K BeadChip in 80 blood samples and >100 samples of tissue for each of the four regions. The second database is ImageCpG, which includes correlations observed in blood, saliva buccal, and brain tissue extracted from epilepsy patients at the University of Iowa [55] assessed with *n* = 12 450K and *n* = 21 EPIC BeadChips (https://han-lab.org/methylation/default/imageCpG). We note that both databases indicate that there is blood/brain correlation for our peak probe in *G0S2* (in ImageCpG *r* = 0.71, *p* = 0.00048, in Essex PFC *r* = 0.31, *p* = 0.0077, and cerebellum *r* = 0.27, *p* = 0.026). However, it must be noted that evidence that cg19534438 is associated with *G0S2* expression in blood or in brain tissue is lacking, and this locus could be involved in regulation of some other gene or simply a biomarker of a PTSD associated process. For example, the iMethyl database (http://imethyl.iwate-megabank.org) [56, 57] of methylation sites and regulatory effects in peripheral blood mononuclear cells (PBMCs) indicates that this locus is regulatory for the adjacent gene *LAMB3* in CD4T cells (effect = 0.0091, *p* = 4.55 × 10^-5^). However, both *LAMB3* and *G0S2* were upregulated in an amyloid β peptide stimulation study of microglia, which suggests that they could be co-regulated [58]. Although we are unable to specifically confirm a regulatory effect for this locus, or confirm association in the brain, cg19534438 and *G0S2* remain an interesting locus for further study in relationship to PTSD based on the strong convergent evidence of association from two independent sources of data and prior studies associating *G0S2* expression to PTSD-related traits.

Methylation at cg05575921 in the *Aryl-hydrocarbon Receptor Repressor* (*AHRR)* gene, which was associated with PTSD in Smith et al. 2019, was among the top 10 results from the Discovery EWAS. In this study and in the consortium meta-analysis, PTSD cases showed reduced methylation at this locus compared to controls. The *AHRR* gene, and cg05575921 in particular, has a well-established association with smoking. The AHR protein is primarily known for its role in xenobiotic metabolism (i.e., metabolism of foreign chemicals generally and aromatic hydrocarbons, specifically). Smoking reduces methylation of *AHRR,* which in turn is associated with increased expression of the gene and enhanced negative feedback inhibition of AHR signaling. DNAm at cg05575921 is one of the strongest and most reliable indicators of smoking in the epigenome [59], but *AHRR* methylation at this locus, and at other CpG sites within the gene, has also been associated with other phenotypes above and beyond the effects of cigarette smoking, including epigenetic age acceleration (e.g., [60]) and C-reactive protein levels (e.g., [61]), both of which have been linked to PTSD in other recent studies [12]. According to iMethyl (accessed Jan 21, 2020), adjacent loci (chr5:373,355 and chr5:373,398) are negatively associated with expression of the *AHRR* gene in monocytes (*p* < 5 × 10^-6^). Additionally, as reported in Smith et al., methylation at this locus is associated with lower kynurenine and kynurenic acid [17]. Based on the examination of the impact of the smoking score and our follow-up analyses using the score and smoking, we conclude that our data are consistent with a PTSD effect which is independent of the association with smoking. However, we note that this will be hard to establish conclusively using human case/control cohorts given the sensitivity of this locus to smoking exposure. For example, even if one were to look at pediatric PTSD in a cohort of children who have never smoked, it is possible that trauma exposure in childhood is associated with increased exposure to second-hand smoke. Therefore, molecular methods (including animal models) may be key in disentangling the association between smoking and *AHRR* methylation from any putative associations with PTSD and/or trauma exposure. The ImageCpG and Essex databases indicate some level of correlation between blood and brain tissue at this locus (in ImageCpG *r* = 0.51, *p* = 0.019; in the Essex database PFC *r* = 0.28, *p* = 0.016, for other regions *p* > 0.05). However, we note that this locus was not associated with smoking in the PFC, and the smoking score was not predictive of a history of smoking in the PFC samples (in dlPFC *p* = 0.44, in vmPFC *p* = 0.38).

When we examined the Consortium Military Cohort replication data, two additional loci from our top 100 associated sites were implicated. These were Amyloid beta A4 precursor protein-binding family A member 1 (*APBA1*) locus cg06826552, through association observed with the 450K BeadChip proxy locus cg13580827, and the Amyloid beta A4 precursor protein-binding family A member 2 (APBA2) locus cg27184903. *APBA1* on chromosome 9 and *APBA2* on chromosome 15 are expressed in neurons and are involved in protein transport and synaptic function [62]. They play a role in the trafficking of APP, and hence potentially play a role in Alzheimer’s disease [62]. *APBA1* (also known as *MINT1* and *X11*) has been implicated in a GWAS of cognitive performance and educational attainment from the UK Biobank [63]. It was also differentially expressed in the brains of schizophrenia cases vs controls [64]. Analyses of genomic deletions have implicated *APBA2* (also known as *MINT2* and *X11L*) in autism [65] and schizophrenia [66] pathogenesis. Teschler et al. observed increased methylation at two *APBA2* CpGs cg21917349 and cg12044210) in female bipolar cases (*n* = 24) compared to controls (*n* = 11) [67]. While *APBA1* and *APBA2* are intriguing PTSD candidate genes, these loci were not genome-wide significant in either the Discovery Cohort or the Consortium Military EWAS, and hence further replication is warranted before these can be considered PTSD loci.

Finally, cg04130728 in the *carbohydrate (chondroitin 4) sulfotransferase 11* gene (*CHST11*) was among the top 10 most strongly associated CpG sites in the Discovery Cohort and this probe associated with PTSD in the PFC at a level that withstood correction for the 100 probes that were examined for replication in the Brain Bank Cohort. CHST11 is an enzyme that is part of a family of carbohydrate sulfotransferases that modify carbohydrate scaffolds involved in mechanisms of extracellular signaling and adhesion. In the brain, CHST11 is involved in generating chondroitin-4-sulfate (C4S), an important component of the brain’s extracellular matrix that regulates neuronal plasticity [68], fear learning [69], and neuroinflammation [70]. The cg04130728 locus is not assessed on the 450K chip, and we could identify no correlated proxies to examine it in the Smith et al. consortium replication data. We were also therefore unable to examine blood/brain correlation in the Essex database. This locus was available in ImageCpG, which did not indicate significant correlation (*r* = 0.11, *p* = 0.62). However, we note that ImageCpG is limited in the number of samples assessed (*n* = 21), is not brain-region specific, and only contains a small proportion of frontal cortex samples. Therefore, it is quite possible that methylation at this probe displays substantial blood/brain correlation in the PFC, but that more data will be needed from specific Brodmann areas before this can be confirmed. As the iMethyl database was generated using 450K data, we were unable to find evidence that this CpG was associated with *CHST11* expression in blood, and we were unable to identify a suitable database for a similar examination of regulatory effects of *CHST11* in brain tissue.

Our examination of candidate probes and gene regions implicated in prior studies of the epigenetics of PTSD replicated the prior observed association with the *AHRR* locus, which was compelling as a candidate locus (*p*_adj_ = 0.00047) despite its lack of genome-wide significance. None of the other previously implicated probes (listed in Additional file 1: Table S2) yielded effect sizes that survived multiple-testing correction. Several factors may explain why *AHRR* was the only locus to replicate. First, our primary analyses adjusted for smoking through the use of a DNAm-based smoking score and this was not done in many of the prior PTSD EWASs. Secondly, our clinical cohorts were comprised exclusively of Veterans, the majority of whom were male and most of whom had chronic PTSD. For this reason, our findings may not generalize to other PTSD populations (and vice versa). It is also noteworthy that 12 of the 51 previously implicated loci had low methylation ranges (range < 0.10; see Additional file 1: Table S6), which could make them unreliable and/or difficult to replicate [45].

## Conclusions

The replication of the *AHRR* locus implicated in the PTSD-consortium EWAS in the discovery cohort, the replication of *G0S2*, the top locus from this EWAS, in the Consortium Military Meta-Analysis, and the broader evidence of agreement in effect size direction for the Discovery Cohort and the Replication Cohort are cause for renewed optimism in the search for reliable blood methylation-based PTSD biomarkers. In many large-scale consortium GWAS studies from the PGC, there was an inflection point past which increasing sample size produced an increasing number of replicable loci. Although it may well be that PTSD DNAm signatures will differ by sex, trauma type, and other demographic factors, these replicated loci may indicate that a similar tipping point has been reached in large sample EWASs of PTSD. Further, this study adds to the growing body of evidence of disruption to inflammation and immune response processes in the pathophysiology of PTSD.

## Supplementary information

**Additional file 1.** Supplementary methods and results.

**Additional file 2.** Complete EWAS results for the Discovery Cohort.

**Additional file 3. **Replication of top EWAS resultsin the PFC of the Brain Bank Cohort.

## Data Availability

A complete set of the results of the EWAS in the TRACTS study, and the results of the top 100 loci examined in the PFC analysis are presented in Additional file 2 and Additional file 3. Due to the provisions of the informed consent documents for this study, individual-level data cannot be posted publicly; however, those data may be obtained via a data-use agreement with the TRACTS study. Contact Dr. Regina McGlinchey (Regina_McGlinchey@hms.harvard.edu) to request access.

## Abbreviations

450K BeadChip: Illumina Infinium HumanMethylation450 BeadChip
CpG: Cytosine-phosphate-guanine site
dlPFC: Dorsolateral prefrontal cortex
DNA: Deoxyribonucleic acid
DNAm: DNA methylation
EPIC: Illumina EPIC Methylation BeadChip
EWAS: Epigenome-wide association study
FDR: False discovery rate
GO: Gene ontology
GWAS: Genome-wide association study
HPA axis: Hypothalamic-pituitary-adrenal axis
*p*: *p*-value
*p*_adj_: False-discovery rate corrected *p*-value
PFC: Prefrontal cortex
PGC: Psychiatric Genomics Consortium
PTSD: Posttraumatic stress disorder
QC: Quality control
SNP: Single-nucleotide polymorphism
TBI: Traumatic brain injury
TRACTS: Translational Research Center for TBI and Stress Disorders
vmPFC: Ventromedial prefrontal cortex
