# Supplementary material for: An epigenome-wide association study of posttraumatic stress disorder in US veterans implicates several new DNA methylation loci
Source: Clin Epigenetics. 2020 Mar 14;12:46. doi: 10.1186/s13148-020-0820-0 (PMC7071645; doi:10.1186/s13148-020-0820-0)
Supplement: Supplementary file 1 — Additional file 1. Supplementary methods and results. [file 13148_2020_820_MOESM1_ESM.pdf]

Supplementary Materials for "An epigenome-wide association study of posttraumatic stress disorder in US veterans implicates several new DNA methylation loci." By M.W. Logue et. al.

Supplementary Methods

Discovery Cohort

The discovery dataset included subjects from the Translational Research Center for TBI and Stress Disorders (TRACTS) cohort, which we have published on previously[1,2]. For this paper, we also included n=37 subjects collected for another study performed at the VA Boston Healthcare System using a similar protocol. Exclusion criteria for both protocols included a history of seizures unrelated to head injury, neurological illness, current diagnosis of schizophrenia, bipolar or other psychotic disorder, severe depression or anxiety, current active homicidal or suicidal ideation, cognitive disorder due to general medical condition other than traumatic brain injury (TBI), and any psychological diagnosis serious enough to interfere with accurate data collection. Subjects completed diagnostic interviews performed by a PhD-level clinician, and an expert consensus team reviewed diagnoses. PTSD diagnosis was based on the Clinician Administered PTSD Scale (CAPS[3]) interview for DSM-IV. PTSD was assessed for three time periods: Pre-deployment PTSD if pre-deployment trauma was present, post-deployment PTSD, and current (last month). DNA samples were processed using the Million Veteran Program (MVP) protocol[4]. EPIC-based methylation data was available for 541 participants. Of those, 513 had complete PTSD-diagnosis and covariate information (age, sex and genome-wide genotype data) and were included in these analyses.

PTSD Brain Bank Cohort

Brains were obtained from the Lieber Institute for Brain Development; clinical characteristics of the Lieber PTSD collection have been described previously in Mighdoll et al. (2018)[5]. Donors were assigned diagnoses based on medical record review and next-of-kin interviews including the MINI International Neuropsychiatric Interview 6.0, the PTSD checklist for DSM-5 adapted for postmortem studies, and the Lieber Psychological Autopsy Interview[5]. In addition, toxicology testing and neuropathological examinations were performed by a board-certified neuropathologist. The cohort was screened to exclude cases with neuritic pathology or evidence of neurodegenerative disease. Smoking status was based on next-of-kin interviews[5].

For the PTSD cases, the average age was 43.01, n=17 were male veterans, n=1 was female veteran. Diagnoses for the n=24 non-veterans (n=19 women) were based on medical records and/or next-of-kin interviews as described above and in further detail in Morrison et al. (2018)[6]. PTSD diagnosis was further assessed by two independent board-certified psychiatrists to arrive at consensus DSM-5[7] PTSD status; each assessor determined a probability score for lifetime PTSD diagnosis on a scale of 1-5; scores of 5 indicated "PTSD definite", 4 indicated "PTSD highly probable", 3 indicated "PTSD probable", 2 indicated "PTSD possible", 1 indicated "PTSD unlikely". PTSD diagnosis was indicated by a score greater than or equal to 3. Of the PTSD cases, n=21 had histories of childhood trauma noted in the medical narratives. Manner of death for the PTSD cases, as determined by the Maryland state medical examiner, included suicide (n=8), undetermined (n=22), accident (n=5), and natural causes (n=7). All n=22 PTSD cases with undetermined manner of death had cause of death related to drug or alcohol use. The average post-mortem interval (PMI) for PTSD cases was 28.87 hours (range 12-48). Although this cohort was screened to exclude individuals with histories of severe TBI exposure, a common PTSD comorbidity, n=6 individuals with PTSD had a history of possible TBI and/or concussion noted in the clinical narratives. For the purposes of this study, controls excluded subjects with a diagnosed mood disorder (MDD, DepNOS, and BP). The n=30 mood-negative controls had an average age of 50.34 years, n=21 were male, average PMI was 29.52 hours, n=3 were veterans, and manner of death was natural (n=21) or accidental (n=9).

Sample Processing

For the Discovery cohort, DNA was isolated from peripheral blood samples using a Qiagen AutoPure instrument with Qiagen reagents. For brain bank tissue, the left hemisphere was received for each subject and was cut into coronal slabs at roughly 0.5 cm intervals while in the fresh state, was photographed, and then snap frozen on aluminum plates and stored at -80°C until tissue extraction. The

coronal slab containing the dlPFC was taken at the level of the genu of the corpus callosum; dlPFC was dissected from Brodmann Area (BA) 9/46. The coronal slab containing the vmPFC was also taken at the level of the genu of the corpus callosum; vmPFC was dissected from BA 12/32. Tissue from each area of interest was dissected and stored at -80°C. DNA extraction was performed using commercially available kits (Qiagen Blood & Cell Culture), and was quantified using PicoGreen dsDNA fluorescent assays (Invitrogen). To determine DNA quality and quantity, TaqMan® RNase P Detection assay (Applied Biosystems Assay, Life Technologies, Carlsbad, CA) with fluorescence detection on a 7900 Fast Real Time PCR System (Applied Biosystems, Life Technologies, Carlsbad, CA) was used.

Genotyping

For the Discovery cohort, DNA samples were hybridized to Illumina HumanOmni2.5-8 BeadChips according to the manufacturer's protocol. Beadchips were imaged using the Illumina iScan System and analyzed with Illumina's GenomeStudio software. Data cleaning was performed using PLINK [8]. X-chromosome genotypes were concordant with self-report sex in all cases. All subject pairs were screened for cryptic relatedness. Self-reported and genetically predicted ancestry was investigated using PC analysis as implemented in EIGENSTRAT [9], and PLINKv1.9[10] based on the genotypes of 100,000 common SNPs.

Genetic variation in the brain-bank cohort was similarly assessed using HumanOmni2.5 chips by the Pharmacogenomics Analysis Laboratory at the Central Arkansas Veterans Healthcare System (PAL). DNA was whole-genome amplified, fragmented, precipitated, and resuspended prior to hybridization on the Illumina HumanOmni 2.5-8 beadchips for 20 hours at 48°C according to manufacturer's instructions. Following hybridization, a multi-layered staining process was conducted, and the Illumina iScan System was used to image beadchips. Results were processed using Illumina GenomeStudio v2011.1 software (Genotyping v1.9.4 module). The resulting data was subjected to the same QC and imputation pipeline as described above.

Methylation Data: Generation and Cleaning

For the Discovery Cohort and Brain Bank cohorts, DNA was quantified using RNaseP Fluorescence on an ABI7900HT Real-Time quantitation system. All samples had sufficient volume, concentration and quality. Zymo EZ-96 DNA Methylation Kits (D5004) were used to bisulfite-convert batched samples; DNA conversion was accomplished via PCR using DAPK1 primers (Zymo) followed by gel electrophoresis of PCR products. Bisulfite-modified DNA was then whole-genome amplified, hybridized to Illumina Infinium EPIC beadchips, single-base extended, and stained using the Automated Protocol for the Illumina Infinium HD Methylation Assay. GenomeStudio Projects were created for each batch for quality control purposes, and chip positions were balanced based on PTSD diagnosis (and brain region for the brain bank data).

Cleaning of the methylation data was performed according to a consortium developed pipeline that we have previously used[1,11]. For the Discovery Cohort, individual-level background-corrected probe data and idat files were output from GenomeStudio. 750 samples were assessed. These include samples from a baseline assessment, plus additional assessments at approximately a 2-year follow-up interval. Cleaning was performed within the CpGassoc package and the ChAMP package in R. The pipeline drops samples that had more than 5% missing data and samples with low intensity (less than 50% of the experiment-wide mean or with intensity <2,000 arbitrary units), but no Discovery Cohort data were dropped due to these criteria. Individual probe values failing to meet a detection p-value threshold of 0.001 were set to missing. Sites with more than 10% missing data (n=1,990 of 865,918 probes) and probes that can cross hybridize between autosomes and sex chromosomes (n=44,051) were excluded, leaving 819,877 probes for analysis. Probe normalization was performed using beta mixture quantile dilation (BMIQ) method as implemented in the wateRmelon [12] R package. Removal of batch and chip effects were performed using an empirical-Bayes batch-correction method (ComBat) as implemented in the Bioconductor sva package[13]. DNA-methylation based sex calls were computed as part of the R code for computing the Horvath DNA methylation age[14] and these were concordant with self-reported sex for all Discovery Cohort samples. We also used the 59 SNP probes on the methylation chip to check IDs for the repeated samples and longitudinal data as well as to check correlation to the genotypes generated by the Illumina HumanOmni2.5-8 BeadChips. We identified 1 sample swap, which was excluded. We additionally dropped redundant methylation values for 11 samples that had been assessed more than once, retaining the methylation data with the lowest missing data frequency and 197 samples

corresponding to repeated measures, leaving methylation data for 541 distinct Discovery Cohort subjects. Of these remaining subjects, 25 were excluded from analysis because they had missing PTSD assessment data and 3 were excluded because they were missing valid genotype data (used to compute ancestry PCs), for a final sample size of n=513 subjects, including n=378 PTSD cases and n=135 controls.

The EPIC-BeadChip data for the Brain Bank Cohort were cleaned using the same pipeline. DNAm data was generated for 3 regions (dlPFC, vmPFC, and motor cortex) together yielding 399 samples. From this, n=2,744 probes were removed because of missing data and 43,957 were removed because they cross hybridized with sex chromosomes. 6 additional samples were removed due to low intensity or high proportion of missing data. Of the remaining 593 samples, 449 were excluded because they were duplicate assessments, from a different brain region, or were non-PTSD subjects with a mood disorder, leaving 144 samples for this study (72 from dlPFC and 72 from vmPFC) from 42 cases and 30 controls.

DNA Methylation Data Analysis.

PTSD association analyses in the Discovery Cohort were performed using a linear model as automated in the Bioconductor Limma package[15]. As is typical for methylation array data, analyses included log 2 logit transformed methylation proportions (M values) as the response, with PTSD as a predictor. Age, sex, and the smoking score (described below) were included as covariates. As DNA methylation patterns tend to vary by ancestry[16] we also adjusted for the first three principal components. Furthermore, as peripheral blood is composed of a heterogeneous mix of cell types, we included covariates for the proportion of white blood cells (CD4 cells, CD8 cells, NK cells, B cells, NK cells, monocytes; the proportion of granulocytes is excluded as the total across all cell proportions sum to 1). These cell counts were estimated from the methylation data using the R minfi package[17,18]. For the EWAS analysis and candidate gene analysis, CpGs with a beta range of less than 0.1 were excluded from consideration after analysis by limma, although these probes were included for replication of previously observed associations. Manhattan plots of EWAS results were generated using the qqman R package. Follow up analyses of the effect of smoking and evaluation of smoking as a confounder were performed using linear models as fit by the R lm package. All follow-up analyses included the same covariates as in the main analyses: age, sex, PCs and white blood cell proportions.

In the Brain Bank data, we used linear mixed model fitted with the lmer function in the lme4 R package. As with the blood-based DNAm analyses, M values were examined as the response with the presence/absence of PTSD as the predictor. This model included age at death and sex as covariates as well as the first 3 PCs for ancestry. Cellular heterogeneity was addressed by including a covariate for the proportion of neurons as estimated from the methylation data using the CETS package[19]. The methylation data from both the dlPFC and the vmPFC were analyzed together in a model that included a random effect for subject and a fixed effect adjusting for region (dlPFC vs vmPFC). The presence or absence of lifetime smoking, which was available for all brain bank subjects, was also included as a covariate. We also performed a genome wide association analysis using limma for the dlPFC and vmPFC separately, so that we could look up significant associations in the joint analysis to determine whether the associations were specific to one region or observed in both regions.

Computation of the DNAm-based Smoking Score.

In the Discovery and Clinical Replication cohorts, we computed a DNAm smoking score using DNAm data based on the weights obtained from a genome-wide examination of smoking and methylation[20]. In that paper, the authors reported effect size estimates for 39 loci that were highly associated with smoking pack years (scaled x100). To generate the smoking score, we computed the product of the M values for these probes times the effect size estimates. In the Discovery Cohort, the resulting score showed highly significant association with both current self-reported smoking status (Cohen's D=1.47, $p<2.2\times10^{-16}$) and with a quantitative self-report measure of daily cigarette usage ($r=0.52$, $p<2.2\times10^{-16}$). Interestingly, this smoking score, which was so strongly associated with smoking in the cohorts with blood samples, was not associated with smoking in the brain-tissue samples from the Brain Bank Cohort (p=0.23 in the dlPFC and p=0.94 in the vmPFC, in a linear model performed in R with age at death, sex, ancestry PCs, and proportion of neurons as covariates).

Supplementary Results

Post-hoc smoking analyses.

Motivated by the persistence of the PTSD-association with the cg05575921 *AHRR* locus in the top 10 probes from the EWAS, we decided to investigate the role of smoking in the top hits and the degree to which the smoking score adjustment adequately accounted for variation in smoking levels. This brings up several questions, the first of which was, "Is cg05575921 exceptional among the smoking associated probes, and in particular the probes used to compute the smoking score?" Supplementary Table 3 presents a series of analyses of the top 10 probes from the score, in order of the strength of association with smoking in the Discovery Cohort (Supplementary Table 3A). The most significant association, as in the Li et al. 2018 smoking EWAS[20], was with cg05575921 ($p=9.18x10^{-33}$) followed by *AHHRR* probe: cg21161138 ($p=3.17x10^{-19}$). We then examined the association of these sites with respect to PTSD, contrasting the results when the score isn't included as a covariate (Supplementary Table 3B) with the analysis when the score is included (Supplementary Table 3C). To our surprise, dropping the score from the analysis of cg05575921 decreased the strength of association observed (0.00081 vs. $9.07x10^{-6}$ in the linear model including the score). However, the resulting effect size estimate differed as expected, that is, the magnitude of the effect size estimate was smaller (less negative) in the adjusted vs the unadjusted model (-0.22 vs -0.13 in the adjusted model). The additional significance in the score-adjusted model is due to the smaller SEs. That is, although there is a reduction in the effect size estimate due to adjusting for smoking, the reduction in the SE in cg05575921 is proportionally greater, and the association between the probe and PTSD was hence more significant. This is not the same pattern we observed with the other smoking associated sites in Table 3, as none of the other smoking associated probes were significantly associated with PTSD after inclusion of the smoking score in the model (Supplementary Table 3C). We also examined the effect of adjusting for self-reported smoking (Supplementary Table 3D). In that analysis, cg05575921 was not significant (p=0.069), as the effect size and sample size were reduced, but the standard error was not reduced to the level observed in the smoking score adjusted analysis. However, when we included both smoking and the smoking score in the analysis (Supplementary Table 3E), cg05575921 was significant (p=0.00059). Finally, to examine the possibility that the increase in significance due to the score adjustment for cg05575921 was partially due to inflation caused by adjusting for a score which included the site in its calculation, we conducted an additional analysis. Specifically, we examined the association between PTSD and methylation at the score loci including a modified score that excluded the particular CpG site being analyzed as a covariate, which we dubbed the $score_{minus}$ (Supplementary Table 3F). That is, the weight for cg05575921 was set to 0 for calculation of the score used as a covariate in the cg05575921 analysis. These new scores were highly correlated with the old scores (all r>0.98). The significance of cg05575921 was increased by this modification ($p=3.91x10^{-6}$), which indicates that the inclusion of cg05575921 in score calculation didn't inflate the significance of the cg05575921 results.

The next question we addressed is, "To what degree are the other top EWAS probes related to smoking?" Supplementary Table 4 presents a series of analyses to address this question. Aside from cg05575921, several other loci from Table 1 were nominally significantly associated with smoking (Supplementary Table 4A). However, these associations are much less significant than the associations observed with PTSD. We also examined the top EWAS loci in a model without the smoking score covariate (Supplementary Table 4B), to examine the effect of inclusion of the smoking score. These analyses indicate that the increased significance of the association between PTSD and methylation when the smoking score was included is unique to cg05575921. For the rest of the loci, the smoking score adjusted and unadjusted results were very similar. Finally, we computed the association between the Table 1 loci and PTSD adjusting for both the smoking score and self-reported smoking (Supplementary Table 4C). In this analysis, despite the decrease in sample size accompanying the inclusion of the smoking variable, all loci remained significantly associated with PTSD. To summarize, our follow-up analyses indicate that the residual association between PTSD and AHRR locus cg05575921 is unique among the smoking associated sites used to calculate the score, and the relationship between cg05575921 and smoking is also unique among the top PTSD-associated hits from the EWAS in the Discovery Cohort.

Post hoc-examination of potential SNP effects.

We examined the sites featured in Tables 1-3 in the original text as well as sites noted in the text from our candidate association analyses. Based on the Illumina annotation, three of these loci had nearby SNPs with > 5% minor allele frequencies: rs3817870 near the *GOS2* locus cg19534438, rs546498991 near the *OR2AG1* locus cg12186981, and rs1550638 and rs1550637 near the *RCCD1* locus cg25526519.

First, we ran post-hoc regression analyses comparing the strength of association between PTSD and log logit transformed methylation at the GOS2 locus with and without rs3817870 in the model (including age, sex, ancestry PCs cell proportions, and the smoking score as covariates in both). Although rs3817870 was associated with cg19534438 methylation (p=0.023), the inclusion of this SNP in the model didn't substantially change the significance or the effect size of PTSD (without rs3817870 beta=0.034 $p=1.25x10^{-7}$, with rs3817870 beta=0.033, $p=3.08x10^{-7}$).

The *OR2AG1* locus cg12186981 noted in Table 3 was one of the top 100 loci from the EWAS analysis, and was featured in Table 3 as it was nominally significant in the brain bank data, but with an opposite direction of effect. There is a SNP nearby, rs471312, which is strongly associated with cg12186981 methylation ($p<2x10^{-16}$). In post-hoc regression analyses, the association between PTSD and cg12186981 was greatly reduced by inclusion of this SNP as a covariate (from $p=8.81x10^{-5}$ to 0.0046). This does not substantially change our interpretation of this locus, which is that it doesn't represent a locus where blood and brain data evince similar associations, as the direction of effect was different.

The *RCCD1* locus cg25526519 was the 6th most significant association from the EWAS. When we compared regression models with and without the nearby SNPs rs1550638 and rs1550637 as covariates we note that while rs1550637 was strongly associated with cg25526519 methylation ($p=5.64x10^{-12}$) the inclusion of these SNPs in the model did not substantially change the estimates of the association with PTSD or its significance (without the SNPs beta= -0.16, p= $9.75x10^{-06}$, with the SNPs beta=-0.17, $p=1.89x10^{-06}$).

Post-hoc Analyses of Potential Confounders:

In regression models with age, sex, ancestry PCs cell proportions, and the smoking score as covariates, we examined the impact of potential confounders: major depressive disorder diagnosis, anti-depressant use, and alcohol use disorder diagnoses on the associations observed between PTSD and the top-10 EWAS loci. Alcohol-use disorders included a diagnoses of alcohol abuse or alcohol dependence, as determined by administration of the Structured Clinical Interview for DSM-IV Disorders[21,22]. Diagnoses of major depressive disorder were also determined by SCID administration. Anti-depressant use was based on self-report. The results of these analyses are presented in Supplementary Table 7. All of the top 10 loci remain significant after inclusion of the covariates. The *GOS2*, *AHRR*, and *CHST11* loci highlighted in this study remain highly significant after the inclusion of potential confounders as covariates (all $p \leq 8x10^{-4}$), and the inclusion of these confounders did not substantively alter the effect size estimates (change in Beta <0.02). The largest observed reduction in significance was for the intergenic CpG cg11504264 whose significance was reduced from $2.15x10^{-6}$ to 0.0018 with the inclusion of MDD diagnosis as a covariate in the model and whose effect size changed from beta=0.24 to beta=0.18. Hence, apart from possibly cg11504264, the significant associations observed in our "top 10" loci do not appear to be a product of confounding with alcohol, depression, or antidepressant usage.

Supplementary Table 1: Demographic Information for the analyzed datasets including the Discovery cohort and the Brain-bank cohort.

Cohort	Chip Used	Specimen	Total N	n Males (%)	mean age (SD)	n Lifetime PTSD cases (%)	n non-PTSD controls (%)	n Veterans (%)	n smokers (%*)	n non-smokers (%*)	n missing smoking info. (%)
Discovery	EPIC	Blood	513	467 (91.03)	32.71 (8.90)	378 (73.68)	135 (26.32)	467 (100)	102 (25.37) based on self-report current smoking	300 (74.63)	111 (21.64)
Brain Bank	EPIC	Tissue from vmPFC and dlPFC	72	43 (59.72)	46.07 (15.04)	42 (58.33)	30 (41.67)	21 (29.17)	32 (44.44) smoking status at time of death based on next of kin interview	40 (55.56)	0%

* % of smokers excludes missing values.

Supplementary Table 2: Candidate Probes and genes examined.

Study	Gene	probe(s)
Smith (2011)	*ACP5*	cg07967308
	ANXA2	cg08081036
	BDNF	cg27351358
	CLEC9A	cg20098659
	TLR8	cg07759587
	TPR	cg24577137
Kuan (2017)	*COL9A3*	cg08696494
	CSMD2	cg06182923
	FAM164A	cg07654569
	intergenic	cg25664402
	PDCD6IP	cg05569176
	TBC1D24	cg09370982
	ZDHHC11	cg05693864
Maddox (2018)	*HDAC4*	cg22937172
Mehta (2017)	*BRSK1*	cg02357741
	DOCK2	cg16277944
	LCN8	cg09325682
	LRRC3B	cg26499155
	NGF	cg17750109
Miller (2018)	*AIM2*	cg10636246
Ressler (2011)	*ADCYAP1R1*	cg27076139
Rutten (2018)	*COL1A2*	cg24406898,cg22676075
	DUSP22	cg03395511, cg18110333, cg21548813, cg01516881, cg11235426
	HIST1H2APS2	cg03517284, cg05785424
	HOOK2	cg06417478, cg11738485,cg04657146
	MYT1L	cg10075506
	NINJ2	cg14911689, cg26654770
	PAX8	cg11763394
	SDK1	cg07249765
Sadeh (2016)	*SKA2*	cg13989295
Uddin 2018	NRG1	cg23637605
	HGS	cg19577098
Smith (2019)	AHRR	cg05575921, cg21161138, cg25648203, cg26703534
	RNF6	cg25415650
	MIR3170	cg17284326
	ATP9A	cg07339236
	AC011899.9	cg26801037
	FLJ46321	cg14405344
	LINC00599	cg18217048
Klengel (2013)	*FKBP5*	NA/ Not assessed on EPIC
Parade (2018)	*HTR2A*	NA/ Not assessed on EPIC
Rusiecki (2013)	*H19*	NA/ Not assessed on EPIC
	IL18	NA/ Not assessed on EPIC
Schecter (2017)	*HTR3A*	NA/ Not assessed on EPIC
Yehuda (2015)	*NR3C1*	NA/ Not assessed on EPIC
Ziegler (2018)	*MAOA*	NA/ Not assessed on EPIC

Supplementary Table 3: Top Smoking-associated Smoking Score probes and their association with Smoking and PTSD in the Discovery cohort.

	A) Association w. Self-reported Smoking			B) Association w. PTSD without controlling for the smoking score or self-report smoking			C) Association w. PTSD controlling for the smoking score		
probe	beta	se	p	beta	se	p	beta	se	p
cg05575921	-0.85	0.065	**9.18E-33**	-0.22	0.066	**0.00081**	-0.13	0.030	**9.07E-06**
cg21161138	-0.30	0.031	**3.17E-19**	-0.048	0.030	0.113	-0.010	0.017	0.55
cg25648203	-0.37	0.042	**3.64E-17**	-0.075	0.039	0.057	-0.038	0.030	0.21
cg01940273	-0.30	0.035	**7.83E-16**	-0.066	0.033	**0.049**	-0.028	0.022	0.20
cg26703534	-0.26	0.032	**1.45E-15**	-0.056	0.029	0.055	-0.027	0.022	0.22
cg03636183	-0.28	0.037	**5.85E-13**	-0.025	0.035	0.48	0.013	0.024	0.58
cg24859433	-0.29	0.044	**6.52E-11**	-0.039	0.040	0.33	-0.00049	0.033	0.99
cg23916896	-0.41	0.065	**6.57E-10**	0.002	0.058	0.98	0.053	0.047	0.26
cg23161492	-0.27	0.046	**9.07E-09**	-0.058	0.041	0.16	-0.025	0.035	0.48
cg07339236	-0.26	0.046	**1.71E-08**	-0.10	0.041	**0.015**	-0.068	0.035	0.052
	D) Association w PTSD controlling for self-report Smoking			E) Association w PTSD controlling for the smoking score and self-report smoking			F) Association w PTSD controlling for $Score_{Minus}$		
probe	beta	se	p	beta	se	p	beta	se	p
cg05575921	-0.12	0.065	0.069	-0.12	0.035	**0.00059**	-0.18	0.038	**3.91E-06**
cg21161138	-0.026	0.032	0.41	-0.027	0.021	0.19	-0.012	0.018	0.52
cg25648203	-0.065	0.042	0.13	-0.065	0.036	0.069	-0.040	0.031	0.20
cg01940273	-0.031	0.035	0.38	-0.032	0.025	0.20	-0.033	0.024	0.16
cg26703534	-0.023	0.032	0.47	-0.024	0.026	0.37	-0.027	0.022	0.22
cg03636183	0.019	0.038	0.62	0.018	0.029	0.54	0.012	0.025	0.62
cg24859433	-0.016	0.044	0.72	-0.016	0.038	0.67	-0.0015	0.034	0.96
cg23916896	0.11	0.065	0.085	0.11	0.054	**0.044**	0.050	0.049	0.31
cg23161492	-0.048	0.047	0.31	-0.048	0.042	0.24	-0.030	0.037	0.42
cg07339236	-0.10	0.046	**0.029**	-0.10	0.041	**0.013**	-0.071	0.036	**0.046**

*Results differ slightly from the EWAS analysis as these do not include the empirical Bayes correction implemented in Limma.

Supplementary Table 4: The top PTSD-associated probes from the EWAS and their associations with self-reported smoking and the DNA methylation-based smoking score in the Discovery Cohort.

**Top PTSD-associated Probes**	**A) Associations with Self-Reported Smoking**			**B) Associations with PTSD without controlling for Self-Reported Smoking Or the smoking score**			**(C) Associations with PTSD with the smoking score and Self-reported smoking in the Model**		
probe	beta	se	p	beta	se	p	beta	se	p
cg19534438	0.015	0.076	0.84	0.33	0.063	**2.99E-07**	0.34	0.075	**7.95E-06**
cg20152234	0.019	0.046	0.68	0.18	0.038	**2.05E-06**	0.19	0.045	**3.18E-05**
cg11504264	0.020	0.059	0.74	0.23	0.050	**6.17E-06**	0.20	0.059	**0.00067**
cg08000207	-0.076	0.093	0.41	-0.35	0.076	**4.99E-06**	-0.39	0.092	**2.65E-05**
**cg05575921**	-0.85	0.065	**9.18E-33**	-0.22	0.066	**0.00081**	-0.12	0.035	**0.00059**
cg25526519	-0.091	0.042	**0.028**	-0.17	0.037	**4.46E-06**	-0.13	0.041	**0.0014**
cg09423651	-0.19	0.14	0.17	-0.53	0.12	**1.16E-05**	-0.58	0.14	**3.41E-05**
cg04130728	-0.006	0.041	0.89	0.15	0.035	**1.21E-05**	0.19	0.040	**2.96E-06**
cg12115116	0.16	0.068	**0.022**	0.25	0.058	**2.63E-05**	0.26	0.067	**9.89E-05**
cg20974659	0.017	0.032	0.60	0.11	0.026	**3.26E-05**	0.11	0.032	**0.00076**

Supplementary Table 5: Association with PTSD for 51 previously reported PTSD candidate loci in the discovery cohort.

Gene	ID	Range	beta	p	p_{adj}
AHRR	cg05575921	0.35	-0.13	**9.16E-06**	**0.00047**
CLEC9A	cg20098659	0.37	0.087	**0.0098**	0.25
BRSK1	cg02357741	0.04	0.054	**0.045**	0.63
ATP9A	cg07339236	0.16	-0.068	**0.052**	0.63
LINC00599	cg18217048	0.20	-0.056	0.062	0.63
TPR	cg24577137	0.028	0.039	0.10	0.69
RNF6	cg25415650	0.056	-0.12	0.12	0.69
AC011899.9	cg26801037	0.19	-0.045	0.16	0.69
LCN8	cg09325682	0.10	0.057	0.17	0.69
ZDHHC11	cg05693864	0.18	-0.12	0.17	0.69
COL1A2	cg24406898	0.32	-0.070	0.17	0.69
SDK1	cg07249765	0.97	-0.51	0.18	0.69
NINJ2	cg26654770	0.93	0.33	0.20	0.69
DOCK2	cg16277944	0.10	0.042	0.21	0.69
AHRR	cg25648203	0.23	-0.038	0.21	0.69
AHRR	cg26703534	0.22	-0.027	0.22	0.69
TLR8	cg07759587	0.51	-0.038	0.27	0.82
NINJ2	cg14911689	0.83	0.18	0.29	0.83
HIST1H2APS2	cg03517284	0.67	-0.089	0.33	0.84
SKA2	cg13989295	0.99	-0.40	0.34	0.84
HGS	cg19577098	0.051	0.028	0.35	0.84
HDAC4	cg22937172	0.047	-0.059	0.39	0.84
HIST1H2APS2	cg05785424	0.50	0.088	0.39	0.84
AIM2	cg10636246	0.25	-0.038	0.40	0.84
HOOK2	cg06417478	0.94	0.23	0.43	0.84
PAX8	cg11763394	0.55	0.076	0.48	0.84
HOOK2	cg11738485	0.97	0.19	0.49	0.84
PDCD6IP	cg05569176	0.18	0.055	0.49	0.84
HOOK2	cg04657146	0.95	0.16	0.52	0.84
MYT1L	cg10075506	0.64	0.067	0.52	0.84
FLJ46321	cg14405344	0.074	-0.025	0.54	0.84
LRRC3B	cg26499155	0.12	0.018	0.55	0.84
AHRR	cg21161138	0.22	-0.010	0.55	0.84
TBC1D24	cg09370982	0.033	0.024	0.56	0.84
COL1A2	cg22676075	0.67	0.052	0.61	0.84
CSMD2	cg06182923	0.31	0.023	0.61	0.84

NGF	cg17750109	0.14	-0.010	0.61	0.84
MIR3170	cg17284326	0.15	-0.010	0.67	0.90
ANXA2	cg08081036	0.044	0.0078	0.70	0.91
DUSP22	cg11235426	0.60	-0.046	0.72	0.91
intergenic-CH1	cg25664402	0.075	0.013	0.75	0.91
COL9A3	cg08696494	0.17	0.0063	0.77	0.91
FAM164A	cg07654569	0.24	0.021	0.78	0.91
DUSP22	cg03395511	0.78	-0.051	0.78	0.91
DUSP22	cg18110333	0.77	-0.038	0.85	0.92
ACP5	cg07967308	0.085	0.0045	0.86	0.92
DUSP22	cg01516881	0.59	-0.028	0.86	0.92
DUSP22	cg21548813	0.68	-0.025	0.89	0.92
BDNF	cg27351358	0.068	-0.0073	0.90	0.92
NRG1	cg23637605	0.21	0.0042	0.92	0.92
ADCYAP1R1	cg27076139	0.039	-0.0036	0.92	0.92

Supplementary Table 6: Association with PTSD for 41 previously reported PTSD candidate genes in the Discovery Cohort. The most significant site per gene is presented as well as a gene-level corrected significance using an FDR adjustment.

Gene	Number of Sites	Most Significant Site per Gene	beta	p	p_{adj}
AHRR	90	cg05575921	-0.13	$9.16x10^{-06}$	0.00082
COL9A3	38	cg08021508	-0.16	0.00066	0.025
CLEC9A	13	cg02930518	0.089	0.0067	0.040
BDNF	31	cg10635145	0.099	0.0025	0.077
NGF	21	cg27181968	-0.16	0.0047	0.098
PAX8	47	cg24409539	0.073	0.0022	0.10
NINJ2	48	cg16639540	0.073	0.0032	0.15
HTR3A	17	cg20621129	-0.089	0.010	0.17
NRG1	124	cg25010216	0.091	0.0039	0.17
ZDHHC11	21	cg15089370	-0.072	0.0082	0.17
HGS	8	cg16549644	-0.085	0.025	0.20
HTR2A	23	cg06020661	0.11	0.015	0.22
SDK1	213	cg22874789	0.20	0.0012	0.25
TPR	2	cg27097224	0.18	0.13	0.26
IL18	11	cg05887493	0.067	0.051	0.29
ANXA2	16	cg21398489	0.079	0.032	0.29
PDCD6IP	6	cg17430014	0.26	0.052	0.31
ATP9A	36	cg05264764	0.25	0.010	0.36
TLR8	9	cg07781550	-0.088	0.12	0.41
AIM2	12	cg17872753	-0.29	0.035	0.41
LRRC3B	17	cg18404374	0.056	0.026	0.44
SKA2	12	cg19273756	-0.055	0.037	0.44
HDAC4	192	cg26443724	-0.092	0.0023	0.45
ACP5	10	cg11417426	-0.035	0.065	0.48
MAOA	24	cg20121427	0.25	0.023	0.50
HOOK2	11	cg07484849	0.11	0.047	0.51
COL1A2	16	cg12563520	0.35	0.059	0.58
ADCYAP1R1	21	cg12140543	-0.046	0.042	0.58
DUSP22	40	cg16305516	0.048	0.021	0.60
BRSK1	7	cg27389454	0.040	0.17	0.66
TBC1D24	17	cg21791024	0.034	0.055	0.67
NR3C1	39	cg00294552	-0.41	0.033	0.68
LCN8	13	cg13764516	0.041	0.11	0.71

FKBP5	30	cg24295963	0.040	0.028	0.73
MYT1L	128	cg02089013	0.065	0.0077	0.74
H19	38	cg17769238	0.031	0.020	0.77
FAM164A	1	cg07654569	0.021	0.78	0.78
DOCK2	50	cg18460239	0.081	0.017	0.80
CSMD2	97	cg07511284	-0.059	0.023	0.81
MIR3170	2	cg03197063	0.030	0.87	0.92
RNF6	7	cg03033508	-0.047	0.17	0.93

Supplementary Table 7: Post-hoc examination of the impact of potential confounders on the association between DNAm and PTSD.

		**Baseline post-hoc analysis**		**Alcohol Use Adjusted Analysis**		**MDD Adjusted Analysis**		**Antidepressant Use Adjusted analysis**	
**Gene**	**ID**	**Beta**	**p**	**Beta**	**p**	**Beta**	**p**	**Beta**	**p**
G0S2	cg19534438	0.34	1.25E-07	0.36	8.46E-07	0.32	1.50E-05	0.34	5.03E-06
BBS9	cg20152234	0.18	1.86E-06	0.19	2.13E-05	0.20	8.48E-06	0.18	5.29E-05
intergenic	cg11504264	0.24	2.15E-06	0.21	0.00032	0.18	0.0018	0.21	0.00052
intergenic	cg08000207	-0.35	6.91E-06	-0.40	7.40E-06	-0.39	2.32E-05	-0.39	2.27E-05
AHRR	cg05575921	-0.13	9.07E-06	-0.14	9.82E-05	-0.12	0.00080	-0.13	0.00037
RCCD1	cg25526519	-0.16	9.75E-06	-0.16	0.00011	-0.16	0.00013	-0.17	7.15E-05
NCK1	cg09423651	-0.53	1.18E-05	-0.55	5.81E-05	-0.59	3.61E-05	-0.62	1.48E-05
CHST11	cg04130728	0.15	1.19E-05	0.17	1.32E-05	0.17	3.37E-05	0.17	6.82E-05
TMLHE	cg12115116	0.25	1.58E-05	0.30	8.42E-06	0.29	2.33E-05	0.28	5.84E-05
intergenic	cg20974659	0.11	1.64E-05	0.11	0.00022	0.11	0.00034	0.13	1.83E-05

Supplementary Figure 1: QQ Plot of the EWAS analysis of PTSD in the Discovery cohort. λ= 1.066.

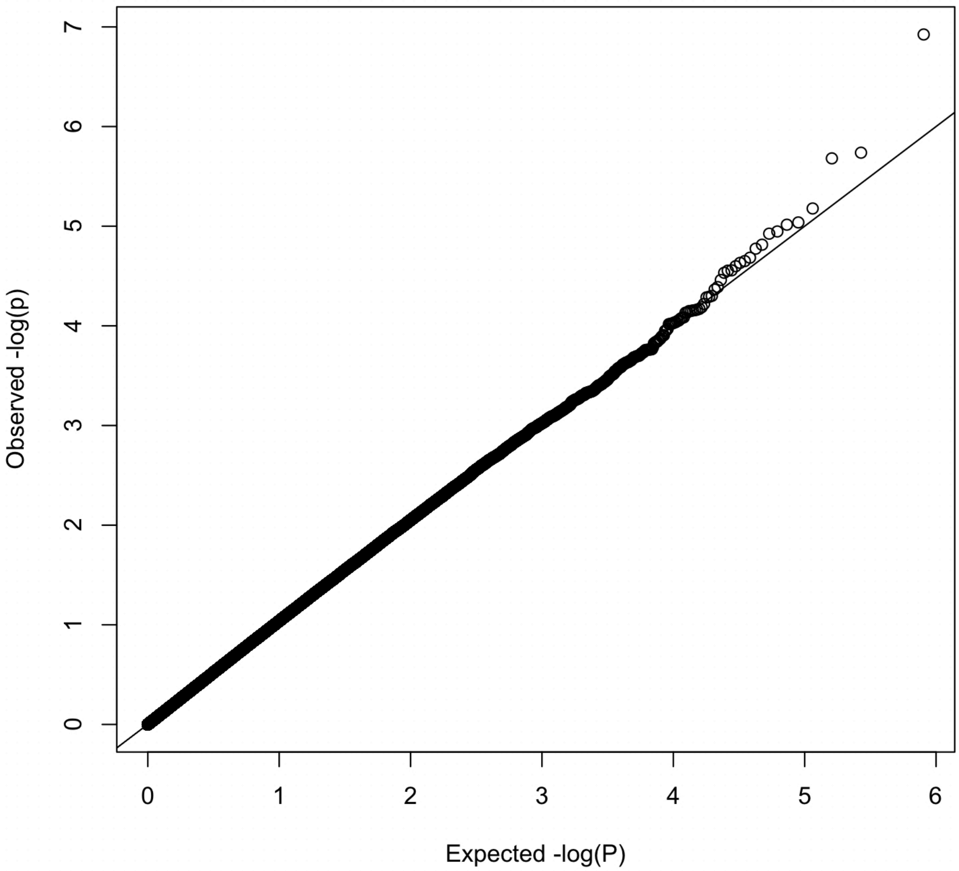

Supplementary Figure 2: Box-scatter plot proportion of methylated DNA (beta) for the top-ten most strongly associated loci from the EWAS of PTSD.

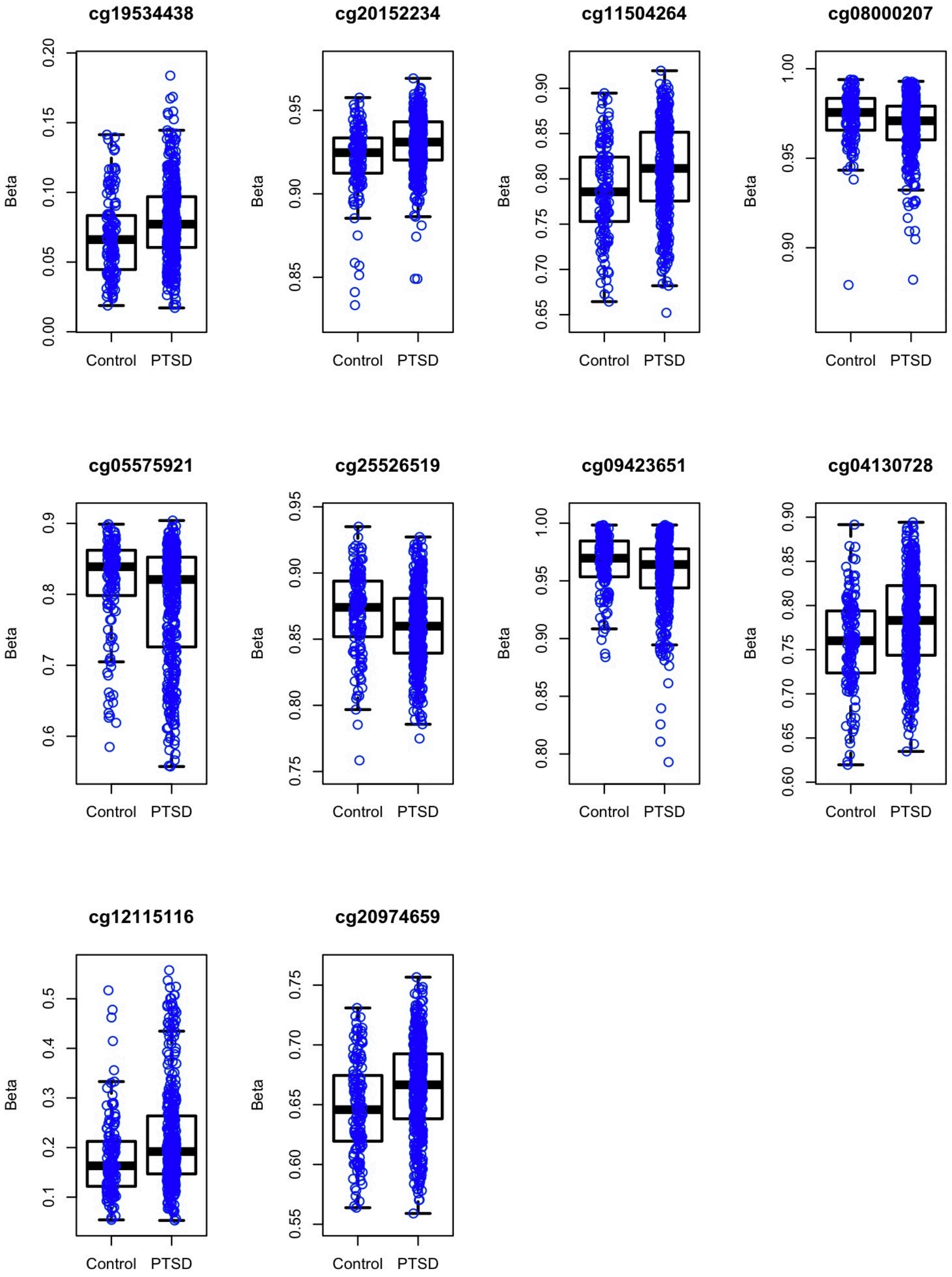

Supplementary Figure 3: Box-scatter plot of the proportion of methylated DNA (beta) for the top EWAS loci which were also nominally (p<0.05) significant in the joint analysis of PFC tissue, with blood, dlPFC and vmPFC methylation plotted separately.

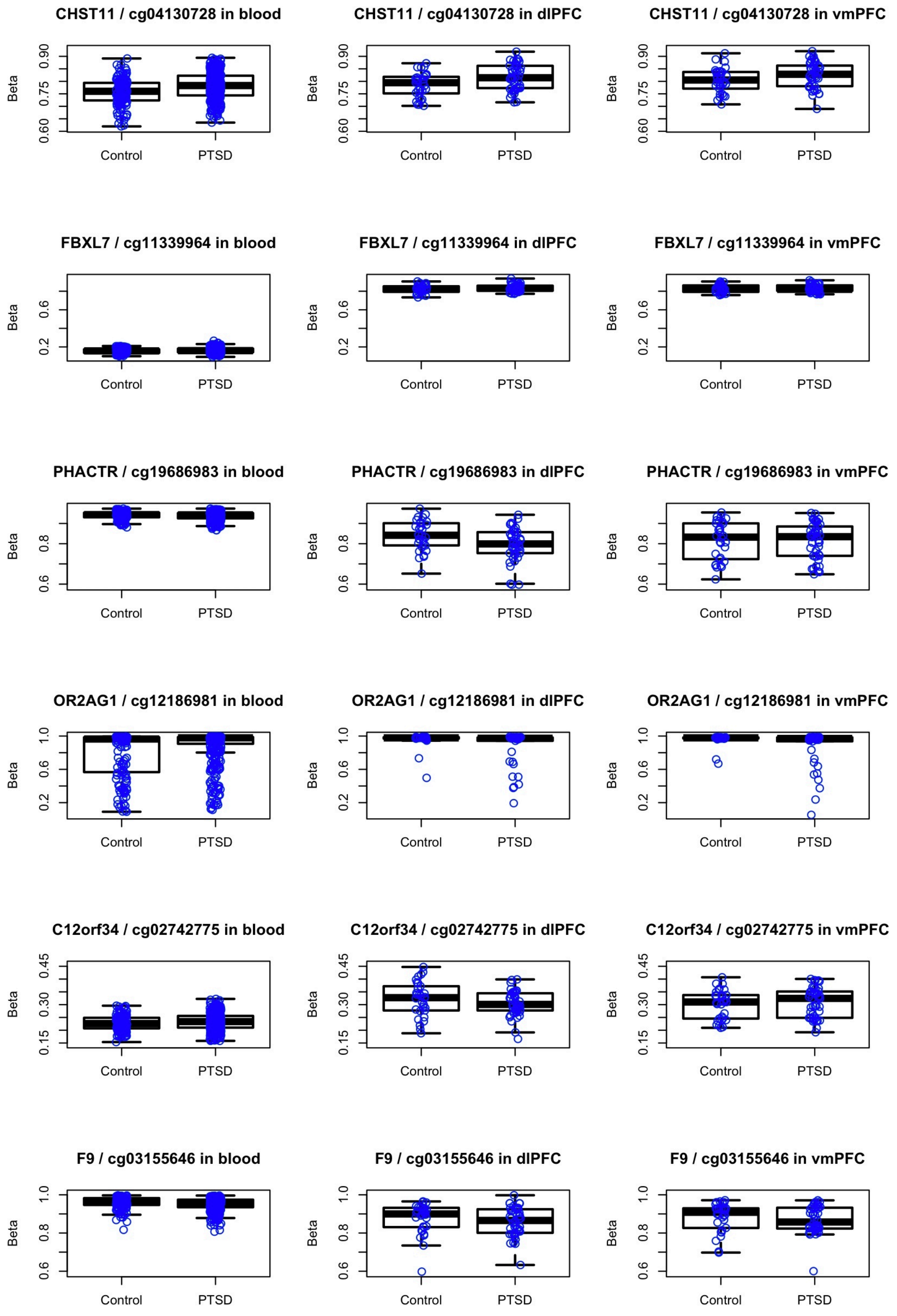

Supplementary Figure 4: QQ Plot of an EWAS analysis of PTSD in the brain bank replication cohort. λ= 0.92.

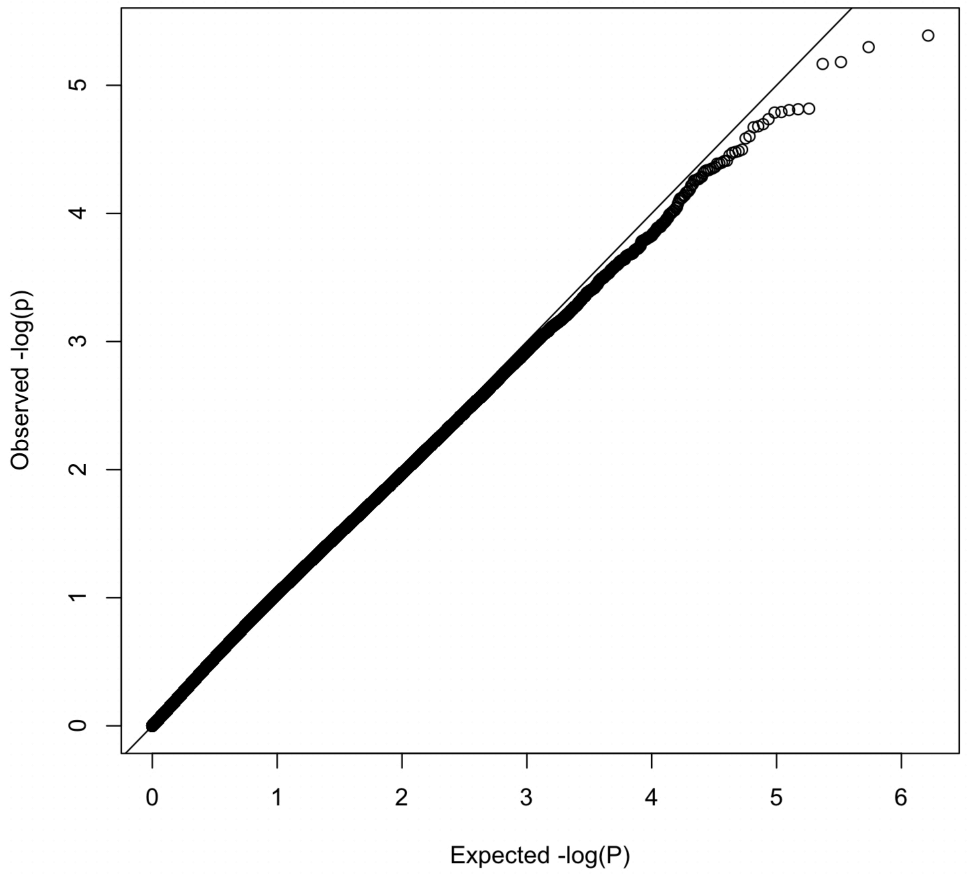

Supplementary References

1. Sadeh, N. *et al.* SKA2 methylation is associated with decreased prefrontal cortical thickness and greater PTSD severity among trauma-exposed veterans. *Mol Psychiatry* **21**, 357-63 (2016).
2. Logue, M.W. *et al.* The correlation of methylation levels measured using Illumina 450K and EPIC BeadChips in blood samples. *Epigenomics* **9**, 1363-1371 (2017).
3. Blake, D.D. *et al. The Clinician-Administered PTSD Scale-IV.*, (National Center for Posttraumatic Stress Disorder, Behavioral Sciences Division., Boston, 1990).
4. Gaziano, J.M. *et al.* Million Veteran Program: A mega-biobank to study genetic influences on health and disease. *J Clin Epidemiol* **70**, 214-23 (2016).
5. Mighdoll, M.I. *et al.* Implementation and clinical characteristics of a posttraumatic stress disorder brain collection. *J Neurosci Res* **96**, 16-20 (2018).
6. Morrison, F.G. *et al.* Reduced interleukin 1A gene expression in the dorsolateral prefrontal cortex of individuals with PTSD and depression. *Neuroscience Letters* (2018).
7. Amercian Psychiatric Association. *Diagnostic and statistical manual of mental disorders (5th ed.).* (American Psychiatric Publishing, Arlington, VA, 2013).
8. Purcell, S. *et al.* PLINK: a tool set for whole-genome association and population-based linkage analyses. *Am J Hum Genet.* **81**, 559-75. Epub 2007 Jul 25. (2007).
9. Price, A.L. *et al.* Principal components analysis corrects for stratification in genome-wide association studies. *Nat Genet.* **38**, 904-9. Epub 2006 Jul 23. (2006).
10. Chang, C.C. *et al.* Second-generation PLINK: rising to the challenge of larger and richer datasets. *GigaScience* **4**, 1-16 (2015).
11. Ratanatharathorn, A. *et al.* Epigenome-wide association of PTSD from heterogeneous cohorts with a common multi-site analysis pipeline. *Am J Med Genet B Neuropsychiatr Genet* **174**, 619-630 (2017).
12. Pidsley, R. *et al.* A data-driven approach to preprocessing Illumina 450K methylation array data. *BMC Genomics* **14**, 293 (2013).
13. Leek, J.T., Johnson, W.E., Parker, H.S., Jaffe, A.E. & Storey, J.D. sva: Surrogate Variable Analysis. R package version 3.10.0.
14. Horvath, S. DNA methylation age of human tissues and cell types. *Genome Biol* **14**, R115 (2013).
15. Ritchie, M.E. *et al.* limma powers differential expression analyses for RNA-sequencing and microarray studies. *Nucleic Acids Res* **43**, e47 (2015).
16. Fraser, H.B., Lam, L.L., Neumann, S.M. & Kobor, M.S. Population-specificity of human DNA methylation. *Genome Biol* **13**, R8 (2012).
17. Aryee, M.J. *et al.* Minfi: a flexible and comprehensive Bioconductor package for the analysis of Infinium DNA methylation microarrays. *Bioinformatics* **30**, 1363-9 (2014).
18. Fortin, J.P., Triche, T.J., Jr. & Hansen, K.D. Preprocessing, normalization and integration of the Illumina HumanMethylationEPIC array with minfi. *Bioinformatics* **33**, 558-560 (2017).
19. Guintivano, J., Aryee, M.J. & Kaminsky, Z.A. A cell epigenotype specific model for the correction of brain cellular heterogeneity bias and its application to age, brain region and major depression. *Epigenetics* **8**, 290-302 (2013).

20. Li, S. *et al.* Causal effect of smoking on DNA methylation in peripheral blood: a twin and family study. *Clin Epigenetics* **10**, 18 (2018).
21. First, M.B., Spitzer, R.L., Gibbon, M. & Williams, J.B. *User's guide for the Structured Clinical Interview for DSM-IV Axis I Disorders SCID-I: Clinician Version.*, (American Psychiatric Publishing, Inc. , Washington, D.C., 1997).
22. Spitzer, R.L., Gibbon, M. & Williams, J.B. *Structured Clinical Interview for DSM-IV Axis I Disorders: Patient Edition (February 1996 Final), SCID-I/P.*, (Biometrics Research, New York State Psychiatric Institute, New York, 1998).
